# Quinolin-2(1*H*)‑one-Based Push–Pull Fluorophores: Tuning Emission from Positive to Inverted Solvatochromism

**DOI:** 10.1021/acsphyschemau.5c00083

**Published:** 2025-12-30

**Authors:** Guillermo E. Quintero, Andrés I. Tello-Soto, Daniela Moraga, Raúl Mera-Adasme, Carolina Aliaga, Margarita E. Aliaga, Moisés Domínguez

**Affiliations:** † Pontificia Universidad Católica de Chile, Facultad de Química y de Farmacia, Escuela de Química, Santiago 7820436, Chile; ‡ 28065 Universidad de Santiago de Chile, Facultad de Química y Biología, Santiago 9170022, Chile; § Universidad de Tarapacá, Facultad de Ciencias, Departamento de Química, Arica 1000, Chile

**Keywords:** fluorescence, inverted emission, solvatochromism, solvatofluorochromism, DFT calculations, quinolin-2(1*H*)-ones

## Abstract

Two donor–acceptor diethylaminoquinolin-2­(1*H*)-ones featuring carbonyl or nitroisoxazole groups as acceptors were synthesized (**DQCh** and **DQI**), and their emissive spectral behavior was recorded in a wide range of solvent polarities. The carbonyl-containing compound nitroisoxazole derivative (**DQI**) represents a newly reported synthesis in this work. Interestingly, the two fluorophores exhibited distinct solvatochromic emission behaviors, with **DQCh** showing a significant bathochromic shift with the increase in solvent polarity, while **DQI** exhibited an unusual inversion in its emission energy at intermediate solvent polarities, transitioning from a bathochromic to a hypsochromic behavior. With multiparametric analysis this unique solvatochromic emission behavior was linked to the different sensibilities of the excited states of **DQCh** and **DQI** to hydrogen bonding from the solvent. These findings position quinolin-2­(1*H*)-ones as a promising scaffold for designing polarity-sensitive emissive dyes, offering new insights into the fundamental understanding and rational design of solvatochromic materials.

## Graphical abstract

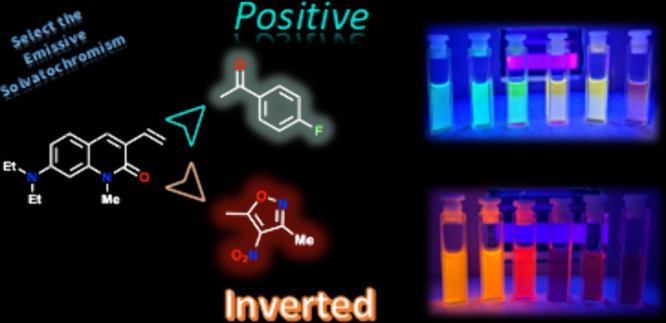

## Introduction

Organic molecules exhibiting intramolecular charge transfer (ICT) have been widely utilized as fluorescent probes,^1,2^ organic light-emitting diodes (OLEDs),^3–5^ bioimaging sensors,^6^ among other imaging applications.^7–10^ An efficient method to promote an ICT state is to conjugate electron donor and electron acceptor groups (EDG and EAG, respectively) in the same structure to promote electronic delocalization through π-system of the resulting molecule.^11^ The presence of the EDG/EAG pair facilitates the ICT process and significantly enhances the change in the dipole moment during the excitation from a less polar ground-state configuration D−π–A to a more polar excited-state configuration D^+^–π–A^–^, or *vice versa*. The distinct polarity inherent to these two states results in a differentiated stabilization through solvent interactions, which induces a pronounced solvatochromic effect on both absorption and emission spectra.^12–14^

A solvatochromic compound is classified as negative, positive, or inverted based on how the energy of its solvent-dependent absorption or emission band changes with increasing solvent polarity.^15,16^ Negative solvatochromic compounds exhibit a gradual shift in their bands toward shorter wavelengths (hypsochromic shifts) as the polarity of the medium increases. In contrast, positive solvatochromic dyes show a progressive change in their bands toward longer wavelengths (bathochromic shifts) with increasing polarity. Finally, inverted solvatochromic dyes transition from positive to negative solvatochromic behavior at a specific polarity value, called the solvatochromic inversion point or SIP.^17–20^ Since the first observation of solvatochromic inversion reported by Jacques in 1983,^21^ various examples of organic dyes that show this behavior have been reported in their solvent-dependent absorption bands.^22–27^ Beyond inverted solvatochromism in organic nonemissive compounds, a few years ago the first case of this phenomenon in organometallic compounds was observed in pyrimidine derivatives, where ferrocene acts as a donor group.^28^ More recently, Morales et al.^19^ reported the first example of emissive inverted solvatochromism by studying a series of aminocarbonyls, suggesting that neutral fluorophores of formula D−π–A capable of forming strong hydrogen bonds in their excited state should exhibit a large change in their transition dipole moment upon excitation compared to nonprotic solvents, triggering an inverted solvatofluorochromic response.^19^

In search of more examples of this novel phenomenon, we focused our attention on the quinolin-2­(1*H*)-one group, a suitable organic scaffold for the synthesis of fascinating fluorescent derivatives.^29–31^ Because quinolin-2­(1*H*)-ones exhibit remarkable photophysical properties,^6^ they could be employed for the design of novel solvatochromic fluorophores in case of introducing electron-donating groups in their structures, such as Et_2_N-, in combination with electron-withdrawing groups, such as carbonyls^7,8^ or electron-poor heterocycles. Therefore, in the present work, we report the emissive solvatochromic behavior of **DQCh** and a new quinoline-2­(1*H*)-one **DQI** (Figure 1), two fluorophores exhibiting quinolin-2­(1*H*)-one as a key component in their π-systems, showing that this moiety facilitates the formation of dipolar ICT states displaying a strong dependence with the change in solvent polarity.

**Figure 1:**
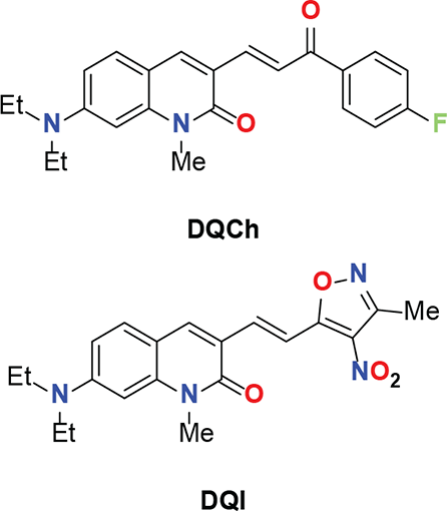
Molecular structures of **DQCh** and **DQI**, the two quinoline-2­(1*H*)-one fluorophores studied in the present work.

## Materials and Methods

### General Procedures

All solvents employed for the spectral measurements were of spectroscopic grade. All reagents were analytically pure and were used without further purification. All reactions were carried out in standard oven-dried glassware. All yields refer to analytically pure samples. NMR spectra were recorded with Bruker Avance 400 MHz equipment. Integrals are in accordance with assignments, and coupling constants are given in Hz. ^13^C NMR spectra are proton decoupled. HRMS analyses were carried out using a Thermo Fisher Scientific Exactive Plus mass spectrometer. The measurements were performed in positive ionization mode under the following conditions: heater temperature, 50 °C; sheath gas flow rate, 5 (arbitrary units); sweep gas flow rate, 0; and spray voltage, 10 kV. Accurate mass determinations were conducted at a resolution of 140,000. Melting points were recorded with a Microthermal capillary melting-point apparatus and were not corrected.

### UV–vis and Emission Measurements

A stock solution of **DQCh** and **DQI** (2 × 10^–5^ mol L^–1^) was prepared in dimethyl sulfoxide (DMSO). From these stocks, 2 μL was dissolved in 2.5 mL of each pure solvent and transferred to 1 cm of quartz cuvettes. The final concentrations of **DQCh** and **DQI** were 2 × 10^–6^ mol L^–1^. Then, the UV–vis spectra were recorded at 25.0 ± 0.1 °C on a Cary 60 UV–visible spectrophotometer. The emission spectra were recorded at 25.0 ± 0.1 °C on a Cary eclipse fluorescence spectrophotometer. The λ_max_^Em^ values obtained in nanometers were used to calculate the electronic transition energies *E*_T_^Em^ in kcal mol^–1^.

### Synthesis of the Fluorophores

**DQCh** was synthesized according to the literature, and all the analytical data matched with those reported.^32^

#### 7-(Diethylamino)­quinolin-2­(1*H*)-one-3-Carbaldehyde (**1**)^12^

Degassed POCl_3_ (27.1 mL, 291 mmol, 7.0 equiv) was introduced into a dry round-bottom flask under a nitrogen atmosphere and cooled in an ice bath. Subsequently, freshly distilled *N*,*N*-dimethylformamide (DMF, 5.6 mL, 72.7 mmol, 3.0 equiv) was added dropwise under stirring, maintaining the reaction temperature between 0 and 5 °C. A solution of 3-(diethylamino)­acetanilide (5.0 g, 24.2 mmol, 1.0 equiv) in 1,4-dioxane (8 mL) was then added slowly while keeping the temperature within the same range. The reaction mixture was then heated to 50 °C and stirred for 20 min. After completion, the mixture was poured portion-wise into a cold saturated aqueous NaHCO_3_ solution (1.5 L) under vigorous stirring. The resulting mixture was neutralized with solid NaOH and crushed ice, maintaining the temperature below 13 °C and adjusting the pH to 5.0. The precipitate formed was collected by filtration, washed with water, extracted with dichloromethane (DCM), dried over Na_2_SO_4_, and concentrated under reduced pressure. The crude semisolid product was purified by column chromatography using *n*-hexane/ethyl acetate (7:3 v/v) as the eluent, affording a dark yellow solid in 27% yield of 2-chloro-7-(diethylamino)­quinoline-3-carbaldehyde. Subsequently, a suspension of 2-chloro-7-(diethylamino)­quinoline-3-carbaldehyde (0.26 g, 1 mmol) in 70% aqueous acetic acid (10 mL) was heated under reflux for 4 h. Upon completion, the reaction mixture was allowed to cool to room temperature, and 20 mL of cold water was added, resulting in the formation of a precipitate. The solid was collected by filtration and washed thoroughly with cold water (5 × 10 mL). The crude product was then purified by column chromatography using dichloromethane/methanol (30:1 v/v) as the eluent, affording **1** as a yellow solid (0.17 g, 71% yield). ^1^H NMR (400 MHz, CDCl_3_): δ = 12.34 (s, 1H), 10.34 (s, 1H), 8.25 (s, 1H), 7.42 (d, *J* = 9.0 Hz, 1H), 6.62 (dd, *J* = 9.0 Hz, 1H), 6.50 (d, *J* = 2.6 Hz, 1H), 3.47 (q, *J* = 7.1 Hz, 4H), 1.25 (t, *J* = 7.1 Hz, 6H). ^13^C NMR (101 MHz, CDCl_3_): δ = 188.96, 164.94, 152.03, 143.97, 141.87, 132.06, 118.83, 110.20, 94.46, 44.85, 12.41. HRMS *m*/*z* [M + H]^+^ calculated for C_14_H_16_N_2_O_2_ 245.1284; found 245.1290.

#### Safety Statements

The synthesis of precursor **1** began with a Vilsmeier–Haack reaction, carried out in a well-ventilated fume hood due to the significant release of hydrogen chloride gas (HCl) upon addition of 3-(diethylamino)­acetanilide to the preformed Vilsmeier reagent. This step must be conducted in a fume hood to avoid inhalation of HCl. The gas evolution was carefully monitored to ensure adequate ventilation throughout the process. All acidic waste generated was neutralized with aqueous sodium hydroxide prior to disposal, following standard safety procedures.

#### 7-(Diethylamino)-1-methylquinolin-2­(1*H*)-one-3-carbaldehyde (**2**)^33^

A suspension of **1** (0.20 g, 0.82 mmol, 1.0 equiv) in DMF (10 mL) was cooled to 0 °C using an ice–water bath. Sodium hydride (60% dispersion in mineral oil, 0.036 g, 0.90 mmol, 1.1 equiv) was added portion-wise under stirring, and the mixture was stirred for 1 h at 0 °C. Methyl iodide (0.056 mL, 0.90 mmol, 1.1 equiv) was then added, and the reaction was stirred for an additional hour at the same temperature. After completion, the DMF was partially removed under reduced pressure, and the residue was diluted with water (30 mL). The aqueous phase was extracted with ethyl acetate (3 × 25 mL). The combined organic layers were dried over anhydrous Na_2_SO_4_, filtered, and concentrated under reduced pressure. The crude product was purified by column chromatography using ethyl acetate as the eluent, affording **2** as a yellow solid (0.196 g, 93% yield). ^1^H NMR (400 MHz, CDCl_3_): δ = 10.37 (s, 1H), 8.20 (s, 1H), 7.47 (d, *J* = 9.0 Hz, 1H), 6.65 (dd, *J* = 8.9 Hz, 1H), 6.29 (d, *J* = 2.4 Hz, 1H), 3.66 (s, 3H), 3.51 (q, *J* = 7.2 Hz, 4H), 1.28 (t, *J* = 7.1 Hz, 6H). ^13^C NMR (101 MHz, CDCl_3_): δ = 189.90, 162.88, 151.93, 144.83, 140.28, 133.42, 118.77, 109.87, 108.94, 93.52, 44.87, 28.74, 12.25. HRMS *m*/*z* [M + H]^+^ calculated for C_15_H_18_N_2_O_2_ 259.1441; found 259.1510 (Figures S1–S6).

#### (*E*)-7-(Diethylamino)-1-methyl-3-(2-(3-methyl-4-nitroisoxazol-5-yl)­vinyl)­quinolin-2­(1*H*)-one (**DQI**)

A solution of 3,5-dimethyl-4-nitroisoxazole (0.0275 g, 0.194 mmol, 1.0 equiv) in ethanol (2.0 mL) was combined with 7-(diethylamino)-1-methylquinolin-2­(1*H*)-one-3-carbaldehyde **2** (0.05 g, 0.194 mmol, 1.0 equiv) and one drop of piperidine at room temperature. The reaction mixture was then heated to 70 °C for 24 h. After this period, the mixture was cooled to room temperature, and the precipitate was filtered and purified by washing several times with diethyl ether (Et_2_O). This process afforded **DQI** as a dark purple solid (0.027 g) with a 37% yield. ^1^H NMR (400 MHz, DMSO-*d*_6_): δ = 8.17 (2H), 7.78 (d, *J* = 16.1 Hz, 1H), 7.54 (d, *J* = 9.0 Hz, 1H), 6.76 (d, *J* = 9.0 Hz, 1H), 6.42 (s, 1H), 3.63 (s, 3H), 3.54 (q, *J* = 7.1 Hz, 4H), 2.5 (s, 3H), 1.22 (t, *J* = 7.1 Hz, 6H). ^13^C NMR (101 MHz, DMSO-*d*_6_): δ = 167.4, 160.2, 155.4, 151.0, 142.3, 141.3, 139.7, 131.3, 126.4, 117.1, 110.1, 108.9, 108.7, 93.7, 43.9 (2C), 28.7, 11.94 (2C), 10.8. HRMS *m*/*z* [M + H]^+^ calculated for C_20_H_22_N_4_O_4_ 383.1714; found 383.1750.

### Computational Details

The conformational freedom of the **DQCh** and **DQI** molecules was studied with the CREST methodology^34^ at the GFN2-xTB level of theory.^35^ As the systems were found to be rather rigid, the top conformer from CREST was optimized, in each case, at the r2SCAN-3c composite density-functional level^36^ using the RI approximation to speed up calculations.^37^ Frequencies were obtained at the same level to verify that each optimized structure was a minimum in the potential energy surface of the respective system. The conductor-like screening model^38^ was employed for modeling solvent effects, using the dielectric constant of toluene (*ε* = 2.38). The density-functional theory optimizations and frequency calculations were performed with Turbomole v7.7.^39,40^

On the minima obtained, energies for Franck–Condon electronic transitions were calculated at the time-dependent density-functional level of theory (TD-DFT) with the TPSSh and def2-TZVPP density functional and basis set, respectively,^41,42^ employing both the RI and the Chain of Spheres approximations for the Coulomb and Exchange integrals, respectively.^37,43^ The conductor polarizable continuum model (CPCM)^44,45^ was used to model solvent effects, with the dielectric constant and refractive index of toluene (*ε* = 2.38 and *n* = 1.5, respectively). The excited state corresponding to the transition with the largest oscillator strength (in both cases, also the one with the lowest energy) was selected for optimization, at the same level of theory, plus the D3 dispersion correction.^46,47^ All the TD-DFT calculations were performed with Orca 6.0.1.^48^

The multiconfigurational character of the ground-state wave function for each system was estimated with the D1 diagnostic^49^ at the MP2/def2-TZVPP level of theory,^41^ implemented in the Turbomole v7.7 package.^39,40^

## Results and Discussion

### Synthesis of the Fluorophores

The **DQI** fluorophore was synthesized in two steps using 7-(diethylamino)­quinolin-2­(1*H*)-one-3-carbaldehyde **1** as the starting material (see Scheme 1). In the first step, compound **1** was *N*-methylated with methyl iodide under basic conditions to obtain **2** with a 93% yield. Finally, quinoline-2­(1*H*)-one derivative **2** was condensed with 3,5-dimethyl-4-nitroisoxazole through Knoevenagel condensation to provide the final product, **DQI**, with a yield of 37%.

**Scheme 1:**
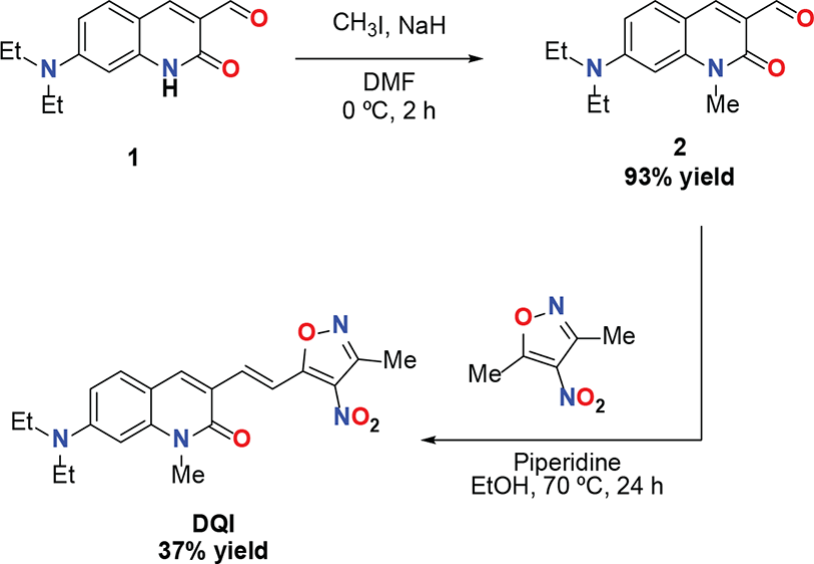
Synthesis of Solvatofluorochromic (*E*)-7-(Diethylamino)-1-methyl-3-(2-(3-methyl-4-nitroisoxazol-5-yl)­vinyl)­quinolin-2­(1*H*)-one **DQI**

Structural characterization was performed using ^1^H, ^13^C (Figures S7–S10), and HRMS spectroscopy (see Figures S11–S12). The Knoevenagel condensation product was confirmed by the presence of an unsaturated double-bond bridge (between quinoline-2­(1*H*)-one and isoxazole moieties), with proton chemical shifts observed at 7.78 ppm (H_α_). Concerning the H_β_, a coupling signal with a three-bonded proton (8.19–8.15 ppm) located in the pyridone moiety of the quinolin-2­(1*H*)-one ring was observed. However, the ^1^H NMR spectra confirmed the (*E*)-isomer of **DQI** based on the coupling constants (*J*) for the β-proton, with a value of 16.1 Hz.

### Emissive Solvatochromic Studies

The steady-state absorption and fluorescence spectra of **DQCh** and **DQI** were recorded in various solvents (complete list in the Supporting Information, see Tables S1–S2 and Figure S13) and spanning a broad range of polarities of *E*_T_^N^: from 1,2-ethylendiol (*E*_T_^N^ = 0.790) as the most polar solvent to cyclohexane (*E*_T_^N^ = 0.006) or toluene (*E*_T_^N^ = 0.099) as the least polar, for **DQCh** and **DQI**, respectively, based on the lower solubility of the first in highly apolar media.

The steady-state absorption spectra of **DQCh** and **DQI** exhibited solvent polarity dependence only in their lower energy bands, occurring at approximately 430–460 and 470–510 nm, respectively. The small spectral variations of their λ_max_^Abs^ (30–40 nm) across the entire polarity range studied result in negligible colorimetric differences of their solution at a macroscopic level. As shown in Figure 2, **DQCh** solutions in toluene (*E*_T_^N^ = 0.099), trichloromethane (*E*_T_^N^ = 0.259), *N*,*N*-dimethylformamide (*E*_T_^N^ = 0.386), 1-octanol (*E*_T_^N^ = 0.537) and methanol (*E*_T_^N^ = 0.762) exhibit comparable yellow tones. In contrast, the emission bands of both dyes showed a notable dependence on the variation in solvent polarity, with spectral shifts of Δλ_max_^Em^ = 126 nm for **DQI** and Δλ_max_^Em^ = 149 nm for **DQCh**, resulting in both cases in macroscopic colorimetric variations. For example, **DQCh** solutions exhibit a spectrum of colors varying from cyan in toluene, light green in trichloromethane, yellow in *N*,*N*-dimethylformamide and 1-octanol, to pale pink in methanol (see Figure 2).

**Figure 2:**
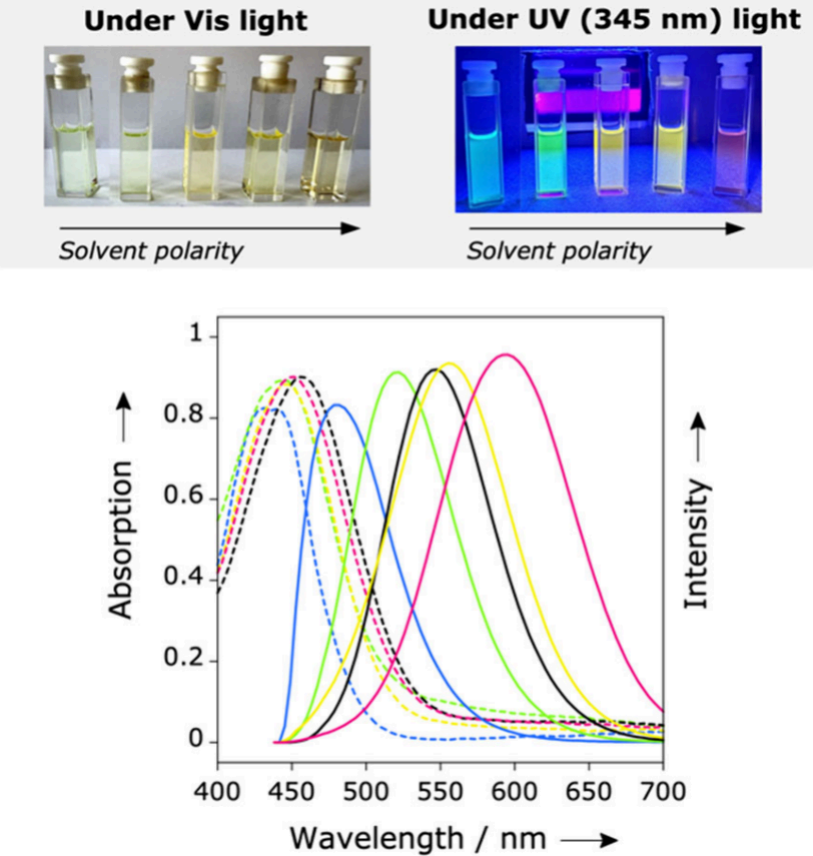
Normalized steady-state absorption and fluorescence spectra of **DQCh** in various solutions of increasing polarity and photographs under UV irradiation at λ = 345 nm. Solvents from left to right: toluene, trichloromethane, *N*,*N*-dimethylformamide, 1-octanol, methanol.

The absorption energy variations of **DQCh** and **DQI** with the solvent polarity *E*_T_^N^ followed rather similar inverted behavior solvatochromic pattern (Figure 3) with solvatochromic inversion occurring at *E*_T_^N^ ≈ 0.45 a solvent polarity close to dimethyl sulfoxide (DMSO). In contrast, the emission energy variation of **DQCh** and **DQI** exhibit distinct solvatochromic behaviors. **DQCh** displays a continuous bathochromic shift in its emission energy as solvent polarity increases across the entire range of recorded polarities, a phenomenon identified as positive solvatofluorochromism. Conversely, the emission energy variation of **DQI** with increasing solvent polarity reveals an inverted solvatofluorochromic behavior, transitioning from a bathochromic to a hypsochromic response at moderate medium polarities (*E*_T_^N^ ≈ 0.444, DMSO).

**Figure 3:**
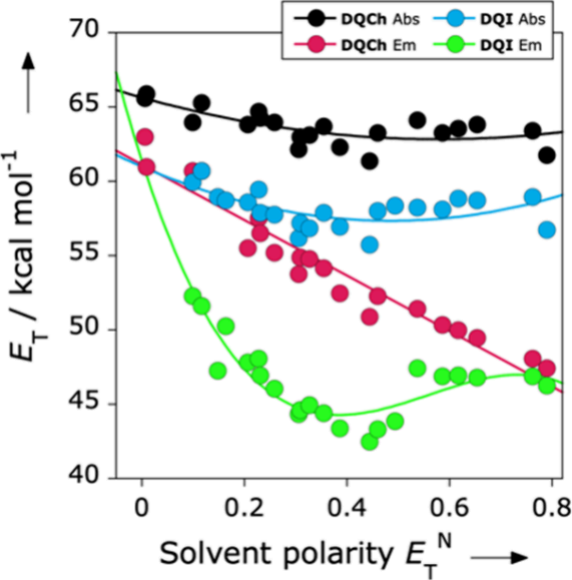
Variations in the absorption *E*_T_^abs^ (black and blue) and emission transition energy *E*_T_^Em^ (red and green) of dyes **DQCh** and **DQI** as functions of the normalized solvent polarity values *E*_T_^N^.

In the nonprotic region (*E*_T_^N^ = 0.006–0.460), where **DQCh** and **DQI** exhibit a positive solvatofluorochromic response, both fluorophores display rather similar sensitivity to changes in solvent polarity, as is evidenced by the similar slope value of their *E*_T_ vs. *E*_T_^N^ curves, with *m* ^Pos^ = −24.6 and −25.1 kcal mol^–1^ for **DQCh** and **DQI**, respectively. This suggests that both fluorophores interact in a similar fashion with the solvent in terms of their excited state stabilization. Nevertheless, this does not seem to be the case in the protic solvent regions (*E*_T_^N^ = 0.460–0.790), where **DQCh** still shows a positive solvatofluorochromic response; however, the donation of hydrogen bonds from the solvent causes **DQI** to invert its emissive behavior from positive to negative solvatofluorochromism,^4^ resulting in a global emission tendency recently revealed and called inverted solvatofluorochromism.

The spectral shifts observed in solvatochromic dyes originate from the differential stabilization that solvents exert on the electronic states of the molecule. In systems showing solvatochromism in absorption, these shifts arise from variations in the relative stabilization of the ground and Franck–Condon excited states. In contrast, emissive solvatochromism reflects the solvent’s influence on the energy gap between the ground and the relaxed excited state. As shown in Figure 3, the solvatochromic response of **DQCh** and **DQI** in absorption is markedly weaker than in emission. This contrast reveals that the ground state of these dyes is relatively insensitive to solvent polarity, whereas their relaxed excited state is strongly affected by it. Consequently, the pronounced solvatochromism observed in the emission spectra can be attributed primarily to the differential stabilization of the relaxed excited state, rather than to changes occurring in the ground or Franck–Condon excited states. It is important to mention that there is no evidence of photodegradation or photoisomerization of **DQI** in different media, thus it couldn′t be involved in the inversion of the emissive solvatochromism, as shown in Figures S15–S18.

To elucidate the influence of solvent effects on the distinct emissive behavior of the **DQCh** and **DQI** fluorophores, their emission energies *E*_T_^Em^ were analyzed across a broad range of solvent polarities using Catalán’s multiparametric approach.^50^ This methodology provides a rigorous framework for quantifying the individual contributions of different solvent–solute interactions in solvatochromic systems.^51^ Within this model, the modulation of a solute property—in this case, the emission energy relative to the gas phase—is expressed as a function of four independent solvent parameters: hydrogen-bond donor acidity (SA), hydrogen-bond acceptor basicity (SB), solvent polarizability (SP), and solvent dipolarity (SdP). Equation 1 summarizes Catalán’s model, where *E*_T_^Em^ denotes the emission energy of the fluorophore in each solvent, and *E*_T_^Em‑0^ corresponds to its emission energy in the gas phase.

1 $${E_{T}}^{\mathrm{Em}}={E_{T}}^{\mathrm{Em}-0}+aSA+bSB+cSP+dSdP$$

Figure 4 illustrates the application of eq 1 to the solvatofluorochromic behavior of **DQCh** and **DQI**. The coefficients for SA, SB, SP, and SdP are represented by blue, pink, green, and orange columns, respectively. A negative coefficient denotes the solvent property’s role in causing a bathochromic shift in the emission band, whereas a positive coefficient signifies its role in a hypsochromic shift.

**Figure 4:**
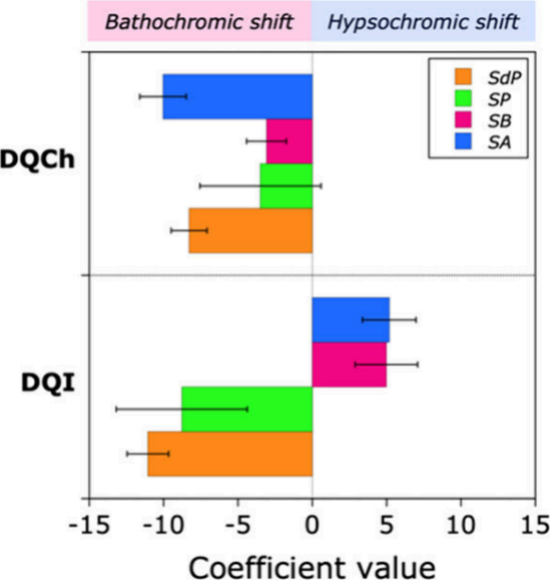
Contribution of Catalán’s solvent acidity (SA, blue), basicity (SB, pink), solvent polarizability (SP, green), and solvent dipolarity (SdP, orange) to the experimental emissive solvatochromism of **DQCh** and **DQI**.

The positive emissive solvatochromism exhibited by **DQCh** is attributable to the stabilization of its excited state via hydrogen bonds (SA) conferred by the solvent, coupled with dipolar interactions (SdP), with contributions from solvent polarizability (SP) and basicity (SB) being negligible. The emissive solvatochromism observed in **DQI** is similarly derived from the same solute–solvent interactions that stabilize the excited state of **DQCh**. Nonetheless, in the case of **DQI**, its positive solvatochromic emissive response in aprotic solvents (*E*_T_^N^ > 0.444) is predominantly due to solvent stabilization through dipolar interactions. As has been observed for other inverted emissive solvatochromic compounds,^4^ the formation of hydrogen bonds by the solvent does not cause higher bathochromic shift but contributes to hypsochromic shift in a slight negative solvatochromic emissive tendency.

In addition to applying the Catalan solvatochromic approach, we also performed a comparative analysis using the Kamlet–Abboud–Taft (KAT) model (Figure S14). While the KAT regression offers a useful empirical framework based on the α, β, and π* parameters,^52^ the Catalan method, with its refined partitioning of solvent polarity and specific interaction terms, yielded a markedly improved correlation with the observed spectral shifts.^51^ In this sense, the KAT model provides results that are qualitatively consistent with those obtained using the Catalan approach, particularly regarding the fluorescence inversion observed for **DQI**, which correlates with α as well as SA and SB contributions within the Catalan framework. However, the Catalan model offers a more precise and physically grounded description of the specific solvent properties that govern emissive behavior across different fluorophores, enabling a more accurate interpretation of how distinct solvation components modulate spectral responses.

### Computational Calculations

DFT calculations were conducted to determine the relative dipolar character of the excited state of **DQCh** and **DQI**. Then, optimization of the excited states at the TPSSh/def2-TZVPP^16,17^ level yields emission energies that correctly reproduce the experimental measurement for the **DQCh** system (Δ*E*_T_^Em^_Exp‑Calc_ = 0.49 kcal mol^–1^) but depart significantly from it for **DQI** (Δ*E*_T_^Em^_Exp‑Calc_ = 29.54 kcal mol^–1^). To explore the causes for the disagreement, we assessed the multiconfigurational character of the ground-state wave function using the D1 diagnostic based on MP2/def2-TZVPP calculations.^24^ The D1 values obtained for both systems (D1_DQCh_ = 0.0558 and D1_DQCh_ = 0.0726) indicate that both systems have a ground state with multiconfigurational character, but that this character is greater for **DQI**, indicating that methods based on a single determinant, such as DFT, are not well suited to describe it. In the Supporting Information, for absorption energies, TD-DFT is also able to reproduce the experimental values for **DQCh**, but not for **DQI**. In a previous work, we proposed an increased degeneracy of the ground state as the reason for inverted and negative solvatochromism in absorption, with increased degeneracy observed when going from positive to inverted and negative systems.^25^ The current data suggests that something similar might occur for the emission behavior. We note that the relatively high multiconfigurational character of the electronic structure of both systems means that higher-level correlated methods, such as CC2 ^53^ would not improve on the TD-DFT description presented, as the systems could only be treated accurately with multiconfigurational methods. These methods’ computational cost scales very rapidly with the ‘active’ orbitals and electrons and it is unfeasible for systems of the size of the dyes studied in this work.

Computational analyses of the excited state of **DQCh** revealed a large redistribution of charge during the emission process. The electron density is highly localized over the 7-(diethylamino)-1-methylquinolin-2­(1*H*)-one subunit in the ground state, while in the excited state, it transitions to a more delocalized distribution across the carbonyl and fluorophenyl groups (Figure 5). Because the relaxed ground state S_0_^R^ of **DQCh** is neutral, light absorption (indicated by blue arrows in Figure 5) promotes a highly dipolar Franck–Condon excited state S_1_^U^. In fact, the difference in the calculated dipole moment values between the two electronic states (μ_e_ = 30.1 D; μ_g_ = 14.5 D) with a corresponding Δμ of 15.6 D implies a highly zwitterionic excited state, being prone to interact by dipolar forces and hydrogen bonding. The corresponding Lippert–Mataga correlations for **DQCh** and **DQI** are provided in Figures S19–S20. Experimentally, the Δμ value was determined through a Lippert–Mataga analysis, affording a value of 16.4 D for **DQCh**, in excellent agreement with our DFT predictions. These findings agree with those presented in Figure 4, in terms of the solvent properties affecting the stabilization of the excited state of **DQCh**. Solvents with low polarity (*E*_T_^N^ = 0.006–0.444) exhibit elevated SdP values, whereas solvents in the polar protic region (*E*_T_^N^ = 0.537–0.762) display high values for both SdP and SA.^26^ These results provide an explanation for the positive emissive solvatochromism of **DQCh**.

**Figure 5:**
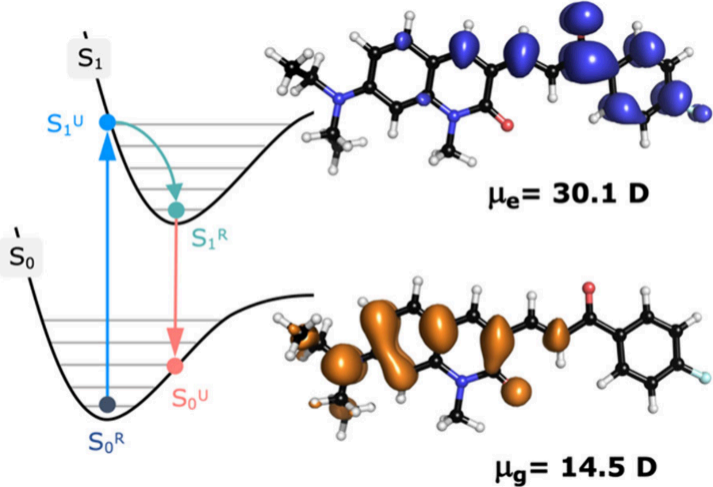
Schematic representation of the S_0_ and S_1_ potential energy surface (PES) of **DQCh** and their electron-density difference distributions for the relaxed excited S_1_^R^ and the unrelaxed ground state S_0_^U^. Calculations performed in toluene solution with the COSMO method^13^ at the TPSSh/def2-TZVPP level of theory.^16,17^

Low polar solvents, such as benzene, toluene, or cyclohexane, exhibit discrete solvent dipolarity values (SdP < 0.270); therefore, once **DQCh** reaches the Franck–Condon state S_1_^U^, this loss dipolarity cannot provide a high energy decrease. Subsequently, the emission energy in these media is considerably greater than that of more polar aprotic solvents. The observed emission energy decreases as the *SdP* increases in conjunction with the solvent polarity *E*_T_^N^ due to a more pronounced stabilization of the excited state of **DQCh** as it transitions from S_1_^U^ to S_1_^R^. Consequently, the progressive decrease in the *E*_T_^Em^ with the increase of *E*_T_^N^ results in the positive emissive solvatochromism observed.

## Conclusions

In this work, the solvatochromic behavior of two newly synthesized 7-(diethylamino)-1-methylquinolin-2­(1*H*)-one derivatives featuring carbonyl (**DQCh**) and nitroisoxazole (**DQI**) electron-acceptor groups, respectively, was thoroughly investigated. These compounds exhibited distinct trends in both absorption and emission spectra across a wide range of solvent polarities. In absorption, both **DQCh** and **DQI** showed a solvatochromic inversion behavior, with the crossover point occurring at moderate solvent polarity (*E*_T_^N^ = 0.444), although the overall spectral shift remained relatively modest Δ*E*_T_^Abs^ = 40 nm. In contrast, their fluorescence responses revealed a much more pronounced solvatochromism, with emission shifts as large as 149 nm. Remarkably, **DQCh** and **DQI** displayed opposite trends in their emissive solvatochromism: **DQCh** followed a positive solvatochromic trend, while **DQI** exhibited inverted emissive solvatochromism.

Multiparametric analysis of the emission profile of **DQCh** and **DQI** revealed their emissive solvatochromism arises from a progressive stabilization by solvent dipolarity accompanying the increase in solvent polarity. Surprisingly, the hydrogen bonding from the solvent contributes to enhancing the positive response of **DQCh** but triggers the solvatochromic inversion of **DQI**. Complementary computational studies supported experimental observations and provided deeper insight into the electronic nature of the excited states of them.

Notably, in this work, the first synthesis of a 7-diethylaminoquinolin-2­(1*H*)-one derivative bearing an isoxazole moiety (**DQI**) is reported, representing a new direction in the molecular design of solvatochromic fluorophores. Interestingly, this compound also exhibits the first documented case of inverted emissive solvatochromism in a quinolin-2­(1*H*)-one-based structure, contributing valuable insight into rare and unconventional photophysical behavior within this versatile scaffold.

## Supplementary Material



## Notes

Additional optical and spectroscopy characterization of **DQCh** and **DQI**, multiparametric regression, and optimized ground-state structures (PDF)
