# Supplementary material for: Quinolin-2(1H)‑one-Based Push–Pull Fluorophores: Tuning Emission from Positive to Inverted Solvatochromism
Source: ACS Phys Chem Au. 2025 Dec 30;6(1):95–102. doi: 10.1021/acsphyschemau.5c00083 (PMC12856668; doi:10.1021/acsphyschemau.5c00083)
Supplement: Supplementary file 1 [file pg5c00083_si_001.pdf]

Supporting Information

Quinolin-2(1*H*)-one-Based Push–Pull Fluorophores: Tuning Emission from Positive to Inverted Solvatochromism

Guillermo E. Quintero,[a] Andrés I. Tello-Soto,[a] Daniela Moraga,[b] Raúl Mera-Adasme,[c] Carolina Aliaga,[b] Margarita E. Aliaga*,[a] and Moisés Domínguez.*,[b]

[a]Facultad de Química y de Farmacia, Escuela de Química, Pontificia Universidad Católica de Chile, Av. Vicuña Mackenna 4860.
[b]Facultad de Química y Biología, Universidad de Santiago de Chile, Av. Bernardo O'Higgins 3363, Santiago, Chile.
[c]Departamento de Química, Facultad de Ciencias, Universidad de Tarapacá, Gral. Velásquez 1775, Arica, Chile.

***E*-mail:** mealiaga@uc.cl (M. E. A.) and moises.dominguez@usach.cl (M. D.)

Contents

1. NMR spectra of novel compounds. S2

2. HRMS spectra of novel compounds. S12

3. Electronic transition energy for the solvatochromic absorption and emission band of DQCh and DQI. S14

4. Steady-state absorption and fluorescence spectra of DQI in various solutions of increasing polarity. S16

5. Multiparametric regressions. S17

6. Photoisomerization studies of DQI. S19

7. XYZ-coordinates for the optimized ground-state structures of DQI and DQCh, and the optimized excited state structure of DQI. S23

8. Solvent effect on the spectral shifts of DQCh and DQI S29

1. NMR spectra of novel compounds.

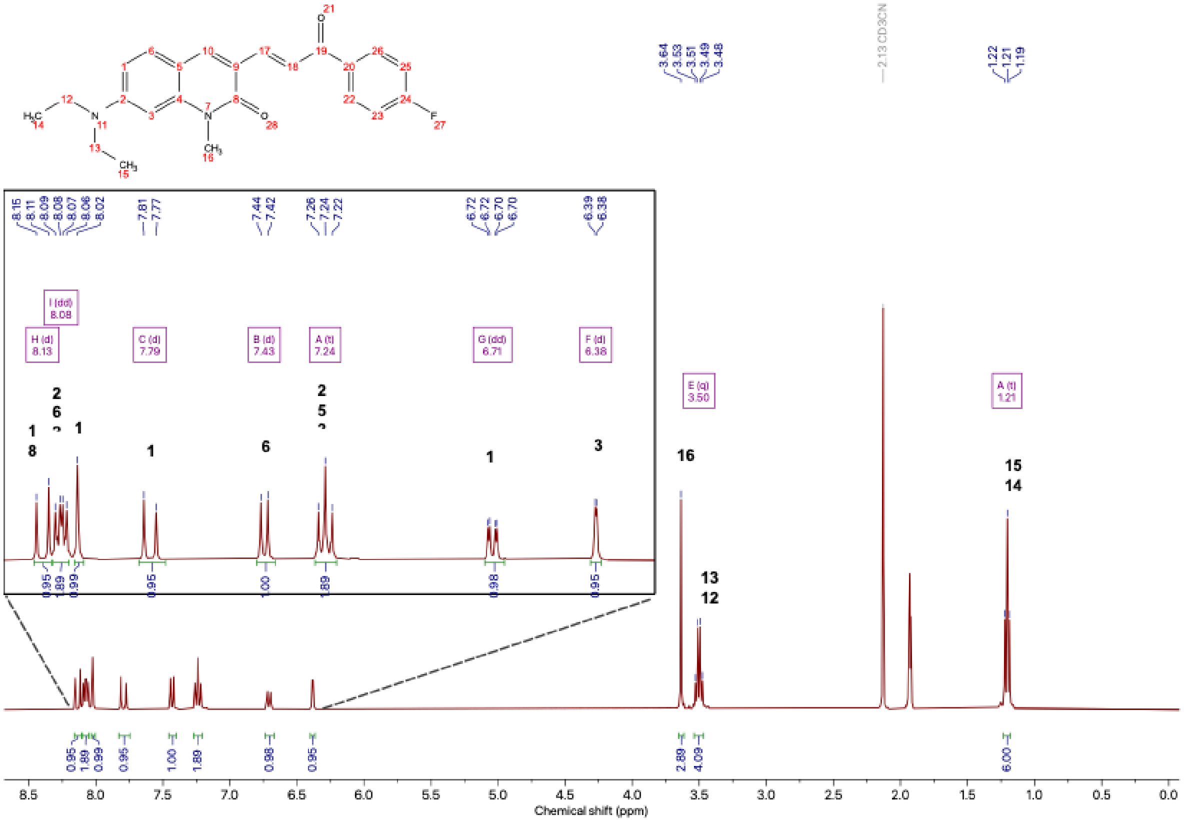

Figure S1. ^{1}H NMR spectrum of the (*E*)-7-(diethylamino)-3-(3-(4-fluorophenyl)-3-oxoprop-1-en-1-yl)-1-methylquinolin-2(1*H*)-one **2**.

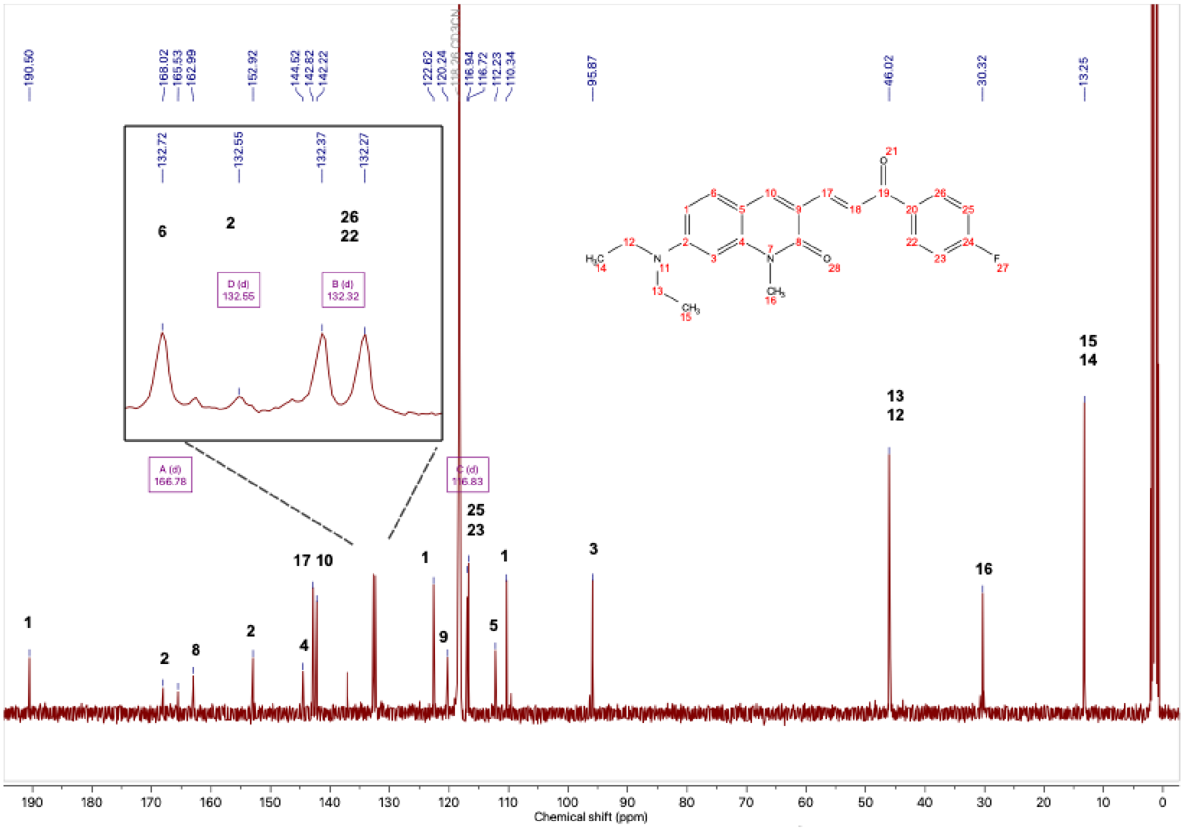

Figure S2. ^{13}C NMR spectrum of the (*E*)-7-(diethylamino)-3-(3-(4-fluorophenyl)-3-oxoprop-1-en-1-yl)-1-methylquinolin-2(1*H*)-one **2**.

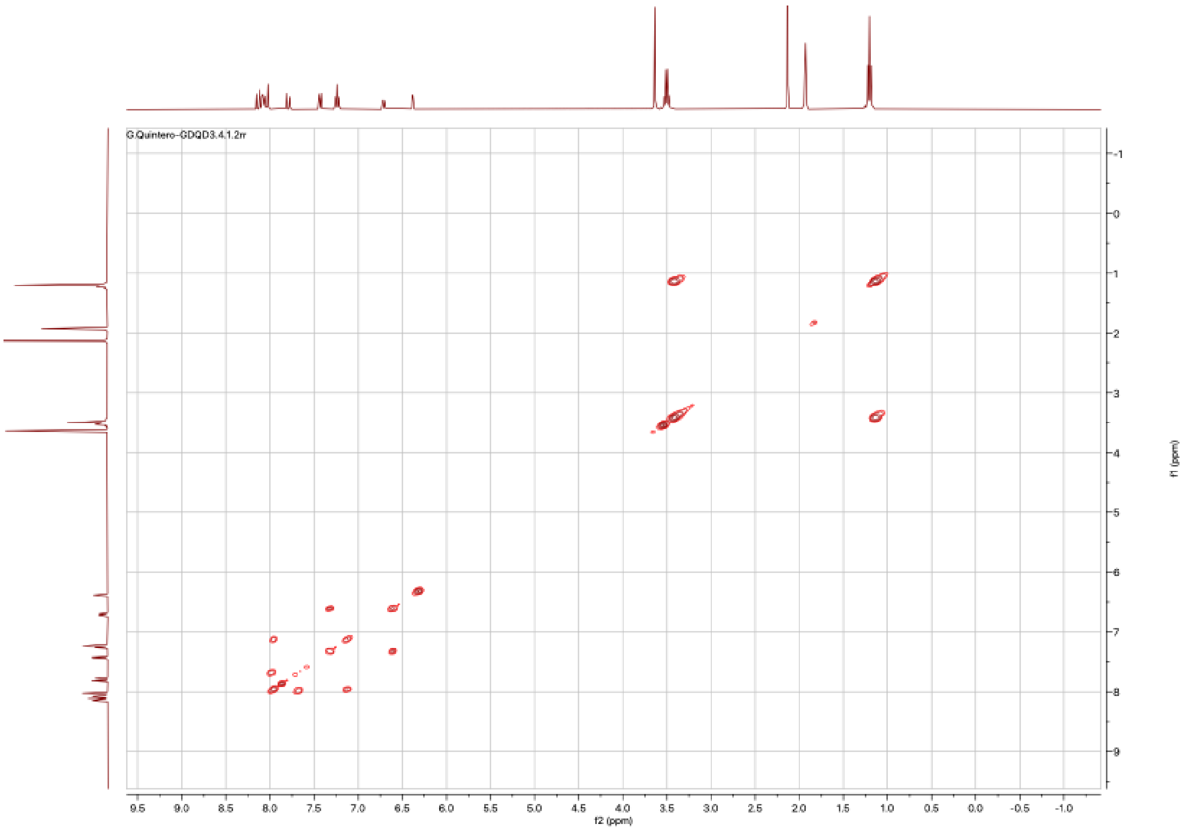

Figure S3. 2D COSY NMR spectrum of the (*E*)-7-(diethylamino)-3-(3-(4-fluorophenyl)-3-oxoprop-1-en-1-yl)-1-methylquinolin-2(1*H*)-one **2**.

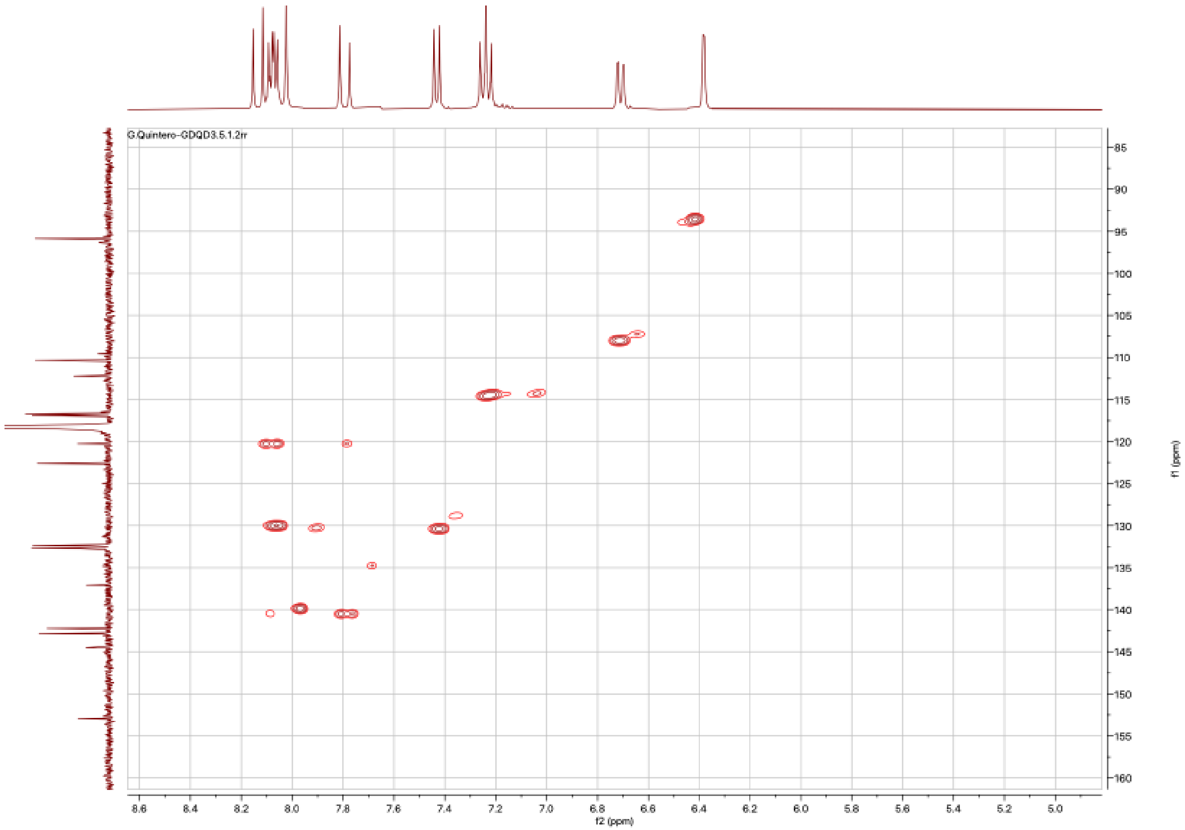

Figure S4. 2D HSQC NMR spectrum of the (*E*)-7-(diethylamino)-3-(3-(4-fluorophenyl)-3-oxoprop-1-en-1-yl)-1-methylquinolin-2(1*H*)-one **2**.

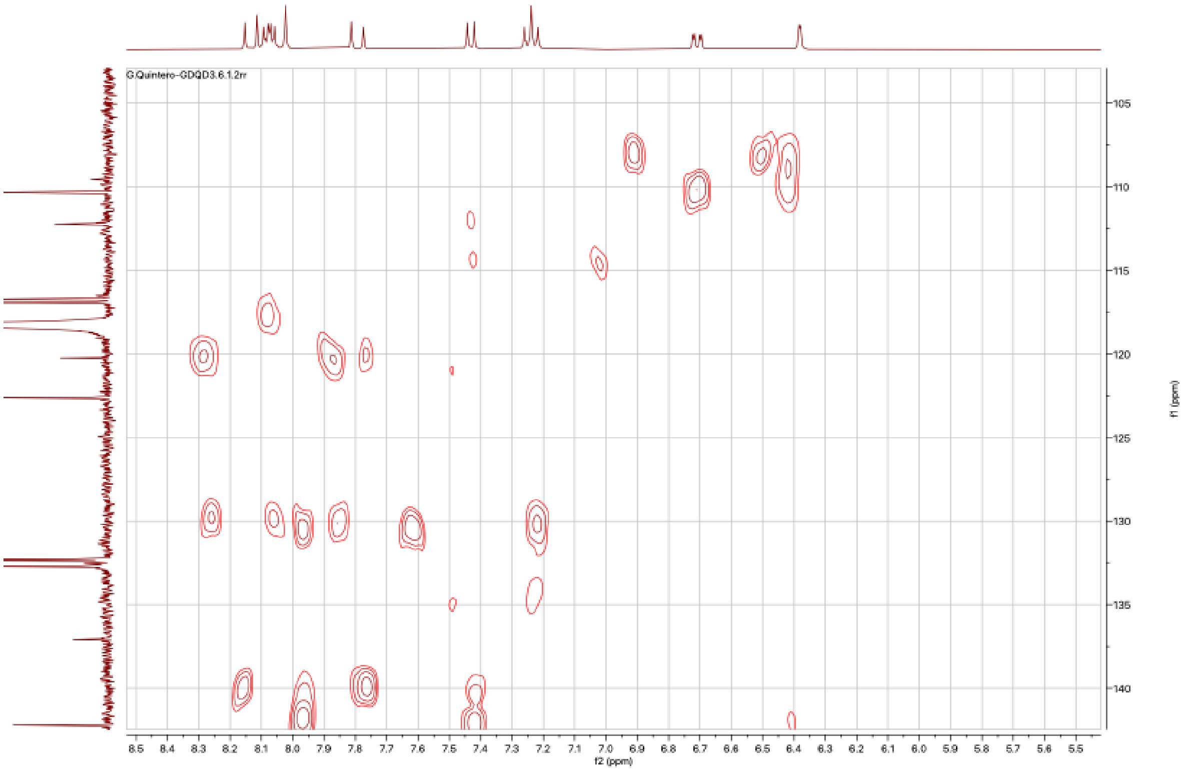

Figure S5. 2D HMBC NMR spectrum of the (*E*)-7-(diethylamino)-3-(3-(4-fluorophenyl)-3-oxoprop-1-en-1-yl)-1-methylquinolin-2(1*H*)-one **2**.

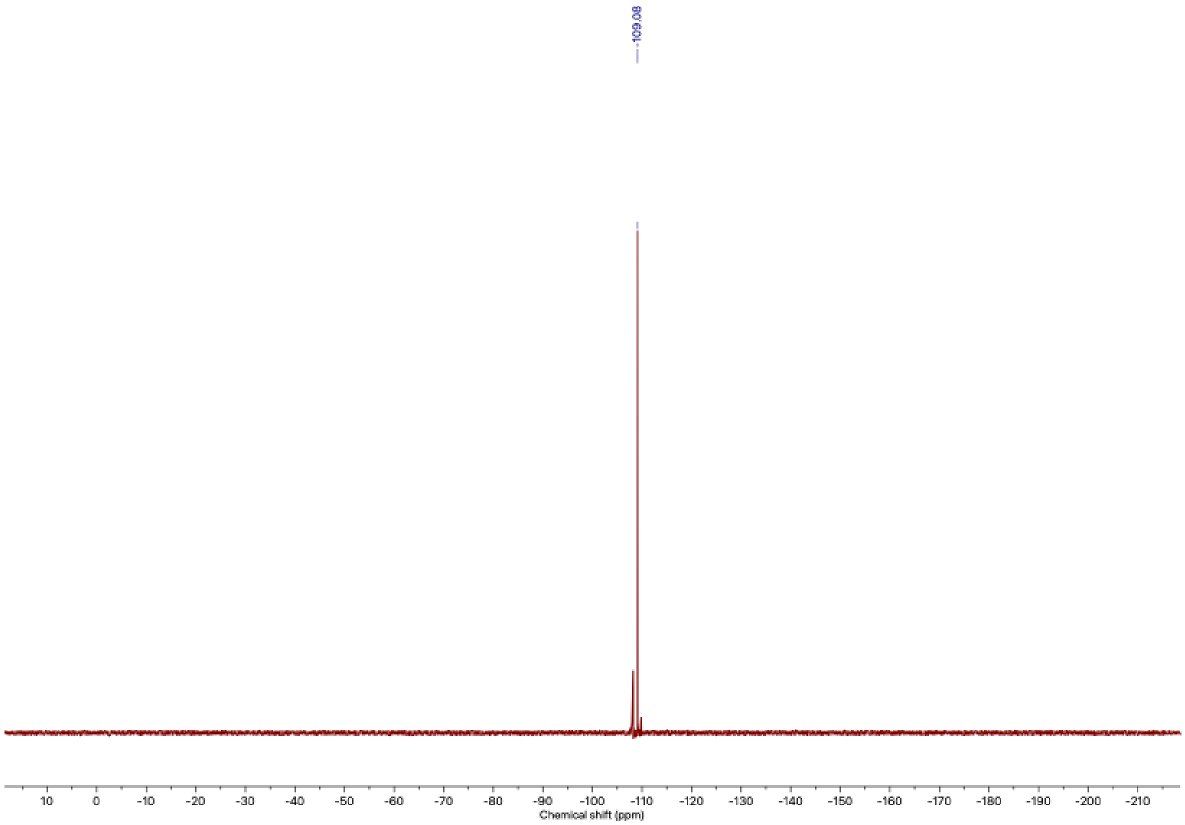

Figure S6. ^{19}F NMR spectrum of the (*E*)-7-(diethylamino)-3-(3-(4-fluorophenyl)-3-oxoprop-1-en-1-yl)-1-methylquinolin-2(1*H*)-one **2**.

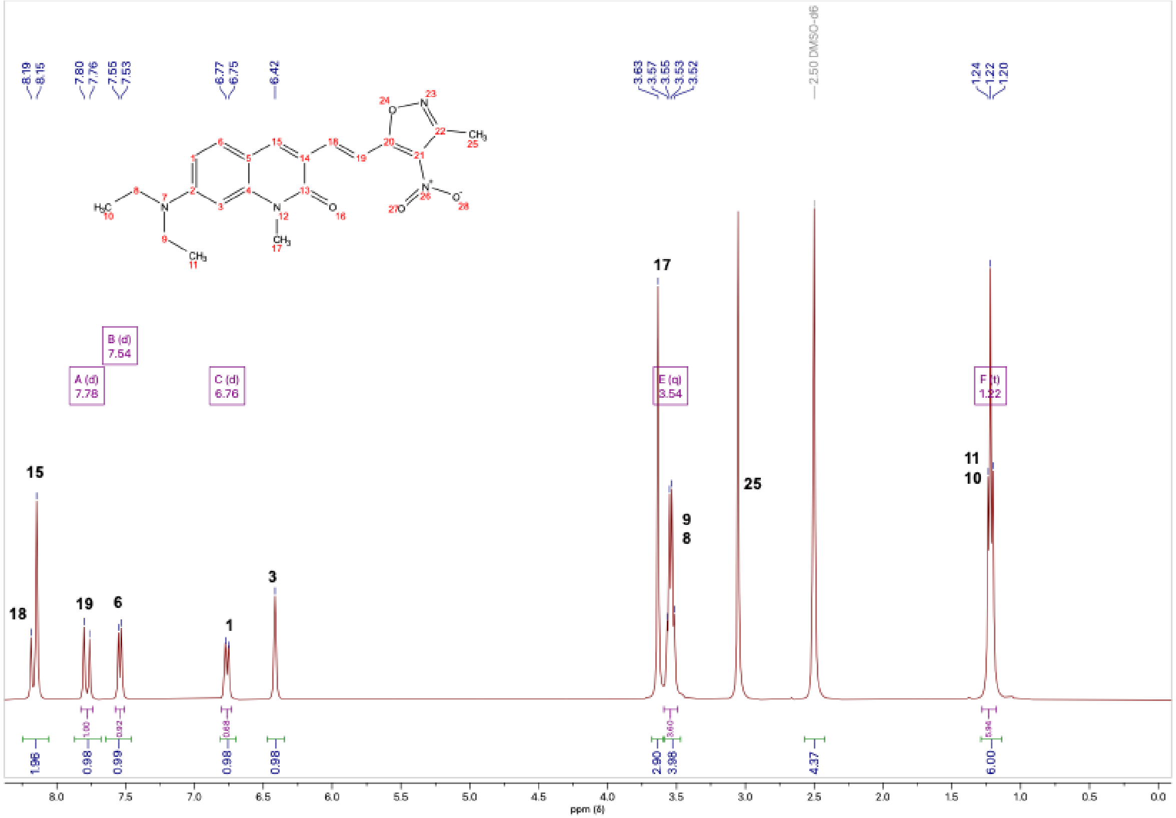

Figure S7. ^{1}H NMR spectrum of the (*E*)-7-(Diethylamino)-1-methyl-3-(2-(3-methyl-4-nitroisoxazol-5-yl)vinyl)quinolin-2(1*H*)-one **DQI**.

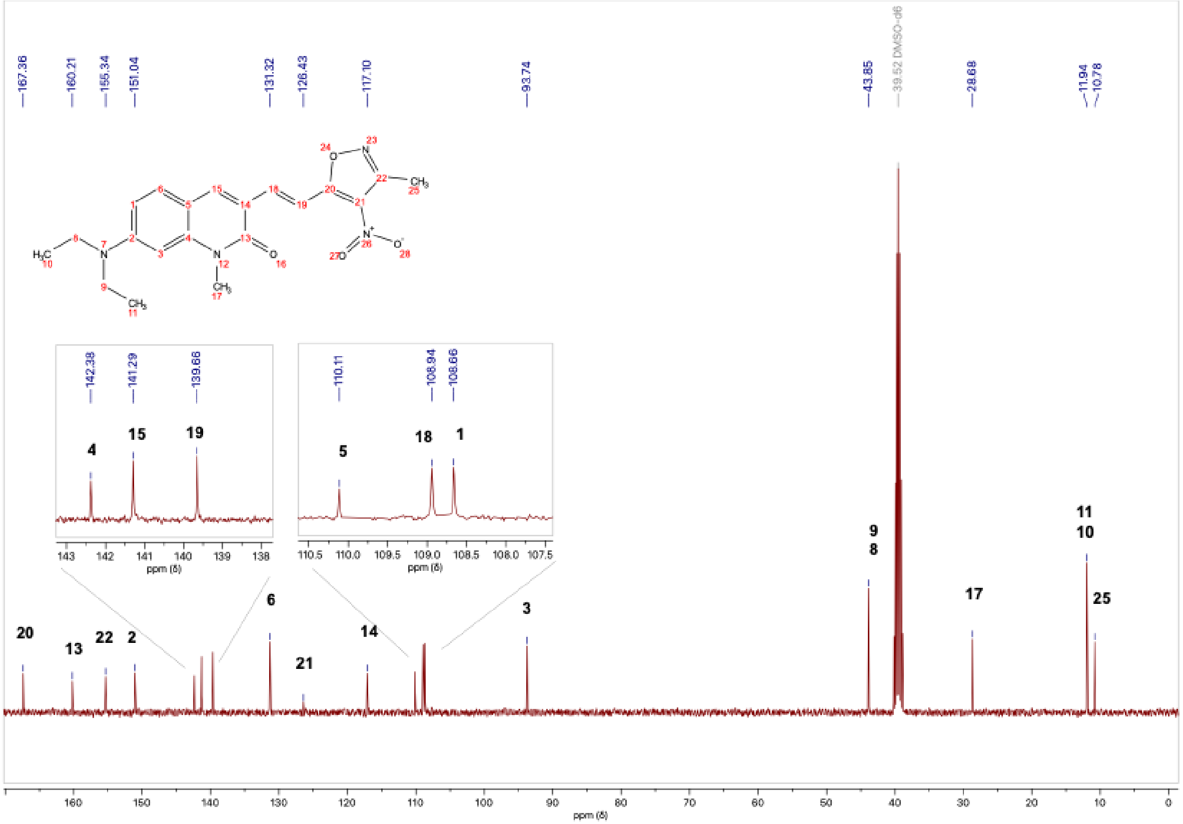

Figure S8. ^{13}C NMR spectrum of the (*E*)-7-(Diethylamino)-1-methyl-3-(2-(3-methyl-4-nitroisoxazol-5-yl)vinyl)quinolin-2(1*H*)-one **DQI**.

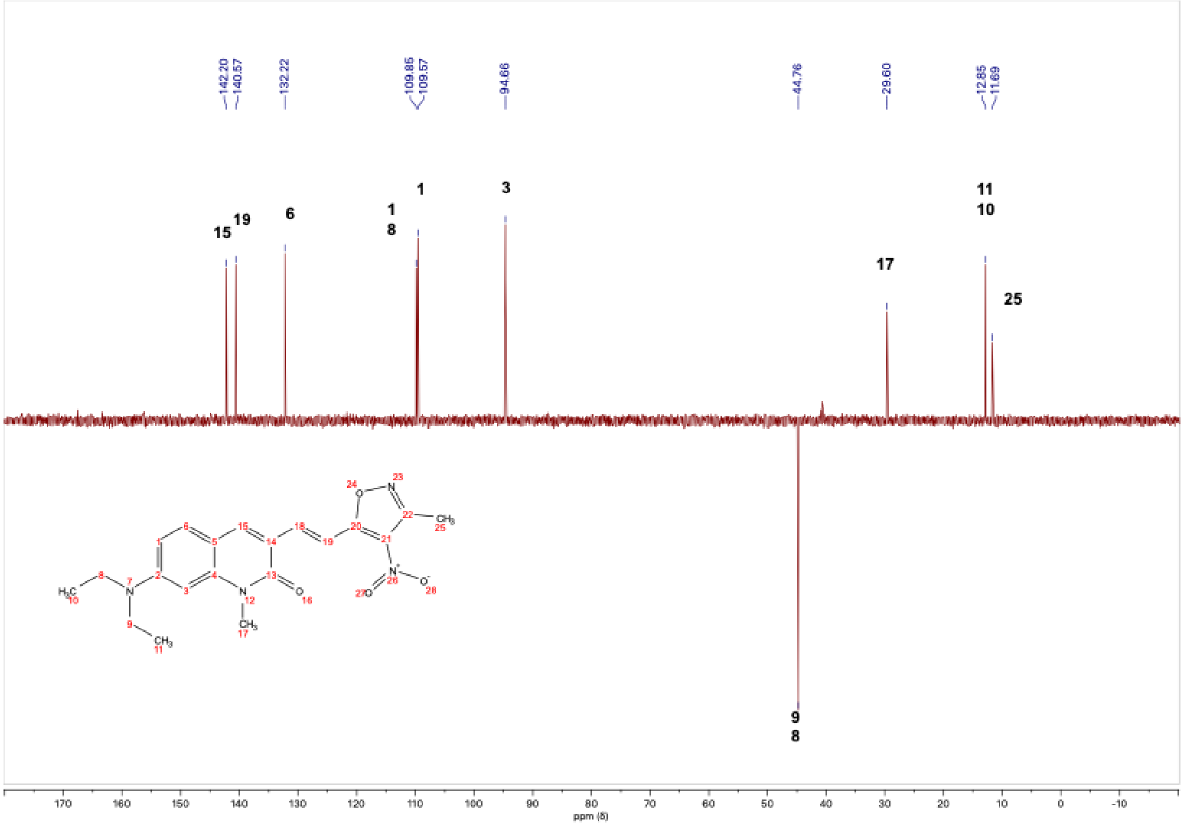

Figure S9. DEPT (Distortionless Enhancement by Polarization Transfer) ^{13}C NMR spectrum of the (*E*)-7-(Diethylamino)-1-methyl-3-(2-(3-methyl-4-nitroisoxazol-5-yl)vinyl)quinolin-2(1*H*)-one **DQI**.

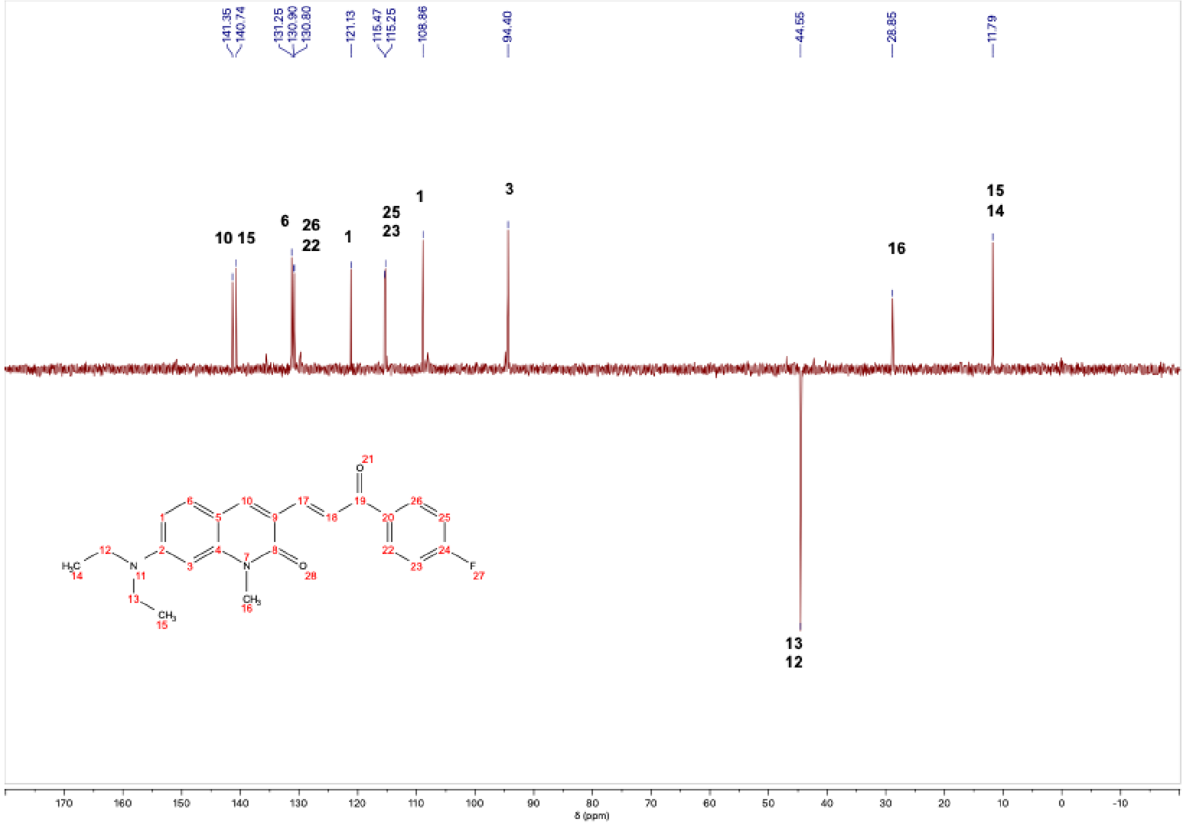

Figure S10. DEPT (Distortionless Enhancement by Polarization Transfer) ^{13}C NMR spectrum of the (*E*)-7-(Diethylamino)-1-methyl-3-(2-(3-methyl-4-nitroisoxazol-5-yl)vinyl)quinolin-2(1*H*)-one **DQI**.

2. HRMS spectra of novel compounds.

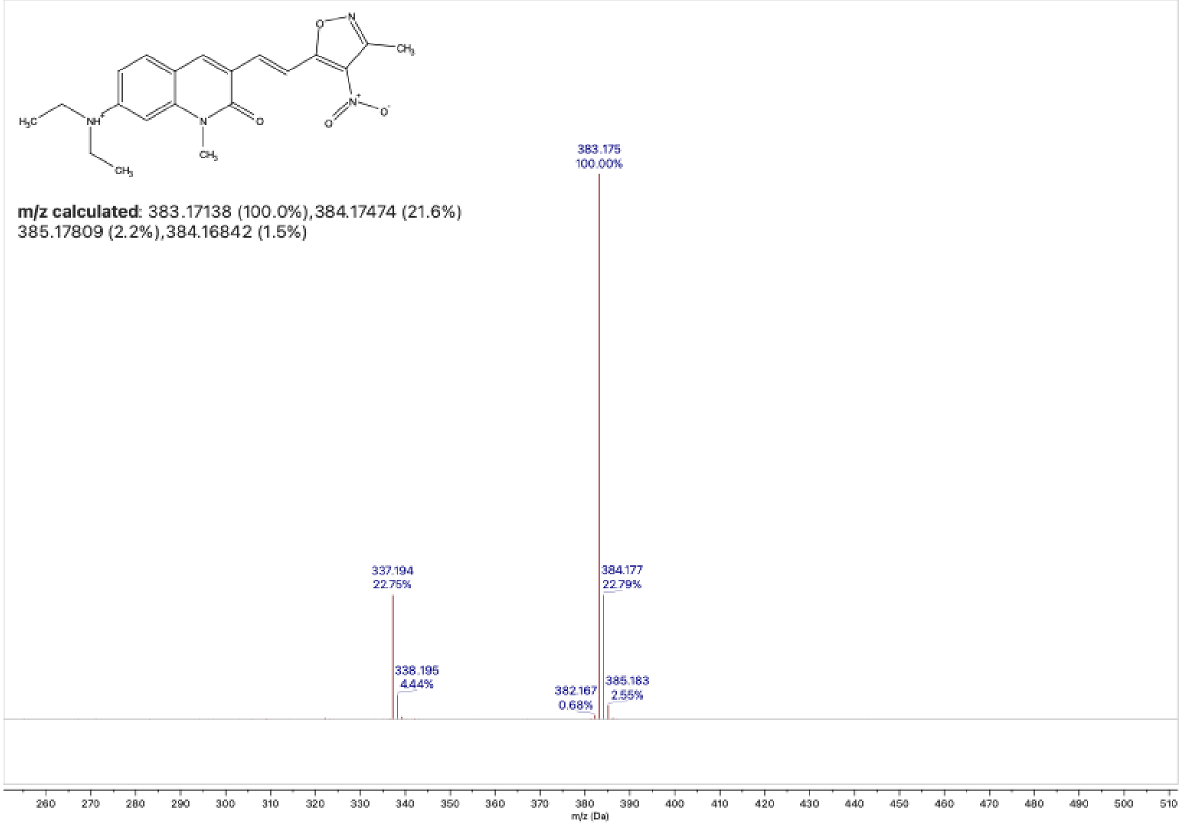

Figure S11. HRMS spectrum of the (*E*)-7-(Diethylamino)-1-methyl-3-(2-(3-methyl-4-nitroisoxazol-5-yl)vinyl)quinolin-2(1*H*)-one **DQI**.

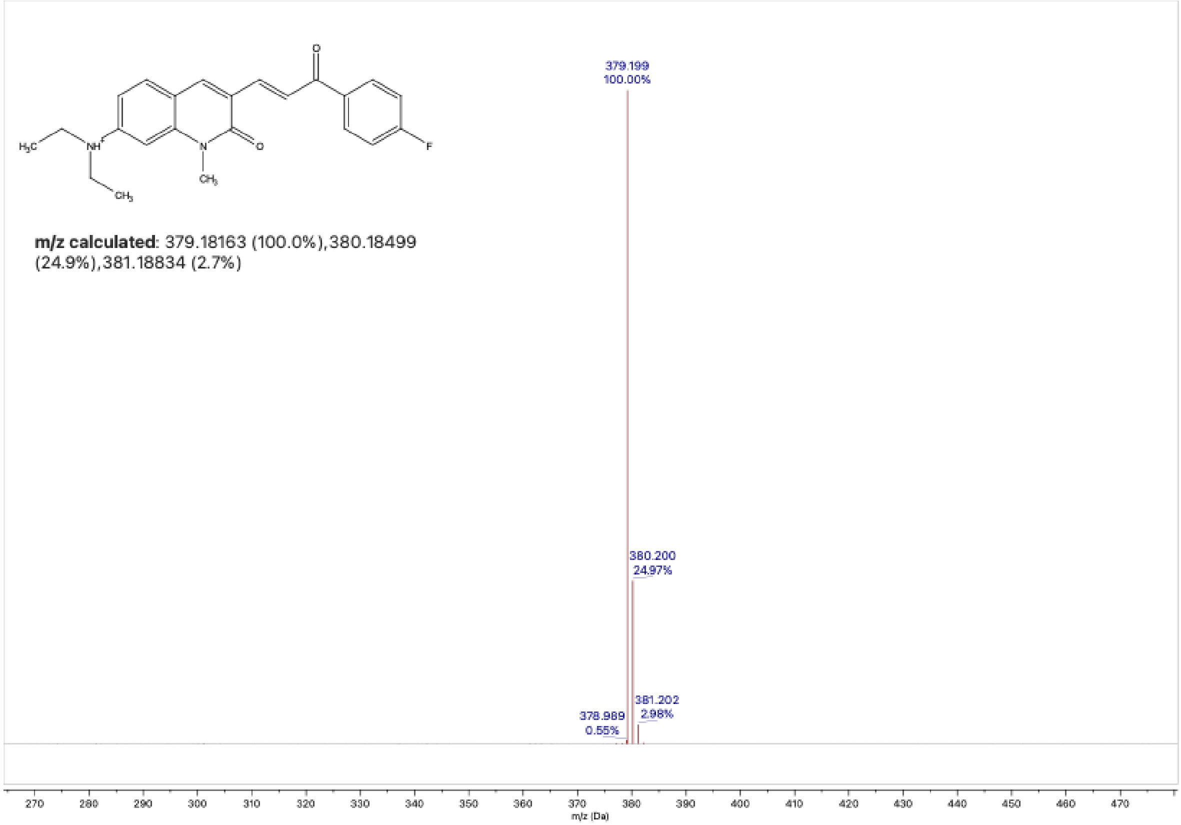

Figure S12. HRMS spectrum of the of the (*E*)-7-(diethylamino)-3-(3-(4-fluorophenyl)-3-oxoprop-1-en-1-yl)-1-methylquinolin-2(1H)-one **DQCh**.

3. Electronic transition energy for the solvatochromic absorption and emission band of DQCh and DQI.

The electronic transition energy of **DQCh** and **DQI** were calculated from the maximum absorption or emission wavelength of their solvatochromic bands. The collection of these E_T values is shown in Table S1–S2.

Table S1. Normalized solvent polarity values E_T^N and electronic transition energies E_T in kcal mol^{-1} of the solvatochromic bands[a] of **DQCh** in various solvents.

Entry	Solvent	E_T^N	E_T Abs.	E_T Em.
1	Cyclohexane	0.006	65.58	62.97
2	*n*-Hexane	0.009	65.88	60.96
3	Toluene	0.099	63.96	60.70
4	Diethyl ether	0.117	65.28	60.70
5	Tetrahydrofuran	0.207	63.82	55.51
6	Ethyl acetate	0.228	64.69	57.53
7	1,2-dimethoxyethane	0.231	64.25	56.50
8	Trichloromethane	0.259	63.96	55.19
9	Acetophenone	0.306	62.15	53.74
10	Dichloromethane	0.309	62.98	54.88
11	1,2-Dichloroethane	0.327	63.11	54.77
12	2-Propanone	0.355	63.68	54.15
13	*N*,*N*-dimethylformamide	0.386	62.29	52.46
14	Dimethyl sulfoxide	0.444	61.35	50.87
15	Acetonitrile	0.460	63.25	52.27
16	Octan-1-ol	0.537	64.11	51.42
17	Butan-1-ol	0.586	63.25	50.33
18	Propan-1-ol	0.617	63.54	49.98
19	Ethanol	0.654	63.82	49.46
20	Methanol	0.762	63.39	48.05
21	Ethane-1,2-diol	0.790	61.75	47.41

[a] E_T calculated from the corresponding λ_{max} values (nm), from the relationship $E_T = 28{,}590/\lambda_{max}$. 1 kcal mol^{-1} = 4.184 kJ mol^{-1}.

Table S2. Normalized solvent polarity values E_T^N and electronic transition energies E_T in kcal mol^{-1} of the solvatochromic bands[a] of **DQI** in various solvents.

Entry	Solvent	E_T^N	E_T Abs.	E_T Em.
1	Toluene	0.099	59.94	52.27
2	Diethyl ether	0.117	60.70	51.61
3	Piperidine	0.148	58.95	47.26
4	1,4-dioxane	0.164	58.71	50.25
5	Tetrahydrofuran	0.207	58.59	47.81
6	Ethyl acetate	0.228	59.44	48.05
7	1,2-dimethoxyethane	0.231	57.88	46.95
8	Trichloromethane	0.259	57.76	46.04
9	Acetophenone	0.306	56.17	44.33
10	Dichloromethane	0.309	57.18	44.60
11	1,2-Dichloroethane	0.327	56.84	44.95
12	2-Propanone	0.355	57.88	44.39
13	*N*,*N*-Dimethylformamide	0.386	56.95	43.38
14	Dimethyl sulfoxide	0.444	55.73	42.48
15	Acetonitrile	0.460	57.99	43.32
16	Acrylonitrile	0.494	58.35	43.85
17	Octan-1-ol	0.537	58.23	47.41
18	Butan-1-ol	0.586	58.11	46.87
19	Propan-1-ol	0.617	58.83	46.95
20	Ethanol	0.654	58.71	46.79
21	Methanol	0.762	58.95	46.87
22	Ethane-1,2-diol	0.790	56.73	46.26

[a] E_T calculated from the corresponding λ_{max} values (nm), from the relationship $E_T = 28{,}590/\lambda_{max}$. 1 kcal mol^{-1} = 4.184 kJ mol^{-1}.

4. Steady-state absorption and fluorescence spectra of DQI in various solutions of increasing polarity.

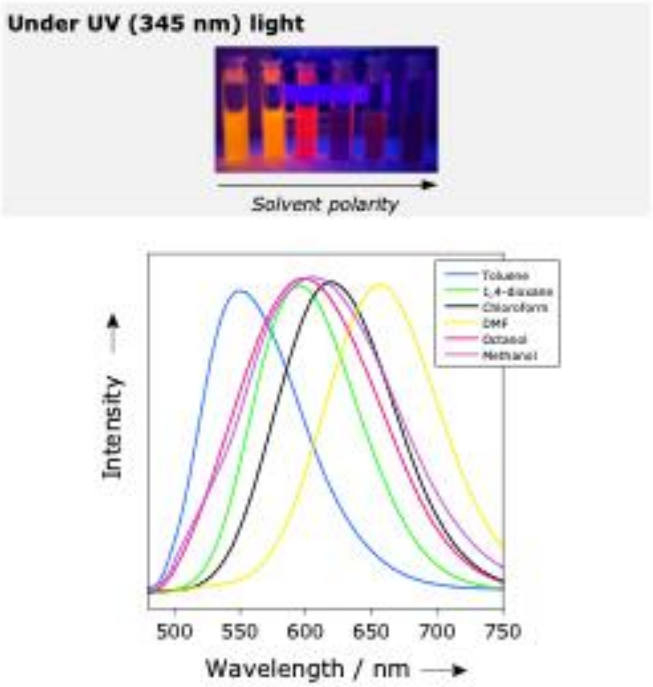

Figure S13. Normalized steady-state absorption and fluorescence spectra of **DQI** in various solutions of increasing polarity, and photographs under UV irradiation at λ = 345 nm. Solvents from left: toluene, 1,4-dioxane, chloroform, *N*,*N*-dimethylformamide, 1-octanol, and methanol.

5. Multiparametric regressions.

The solute-solvent interactions responsible for the emissive solvatochromism of **DQCh** and **DQI** were quantified using a multiparametric regression analysis with the Catalan's scales for solvent hydrogen-bond donor (HBD) acidity (*SA*), hydrogen-bond acceptor (HBA) basicity (*SB*), solvent polarizability (*SP*), and solvent dipolarity (*SdP*).

$$E_T(\text{fluorophore}) = E_T^0 + aSA + bSB + cSP + dSdP \quad (1)$$

The values of the regression coefficients a, b, c, and d represent the relative importance of the four solvent properties on the solvatochromic response of **DQCh** and **DQI**, and the sign of these coefficients indicates their contribution to negative (positive coefficient values) or positive (negative coefficient values) solvatochromic response. The results obtained are shown in Tables **S3**–**S5**.

Table S3. Coefficients obtained for the Catalán multiparametric regressions for the positive emissive solvatochromism displayed by **DQCh**.

Parameter	Value
E_T^0	65.00 ± 2.93 kcal mol^{-1}
a	-10.04 ± 1.56
b	-3.08 ± 1.34
c	-3.49 ± 4.07
d	-8.29 ± 1.21
r^2	0.930

Table S4. Coefficients obtained for the Catalán multiparametric regressions for the positive emissive solvatochromism displayed by **DQI**.

Parameter	Value
E_T^0	60.42 ± 3.24 kcal mol^{-1}
a	–[a]
b	–[a]
c	-8.7 ± 4.40
d	-11.06 ± 1.40
r^2	0.875

[a] Discarded parameter during the regression for not contributing to the positive emissive response of **DQI**.

Table S5. Coefficients obtained for the Catalán multiparametric regressions for the negative emissive solvatochromism displayed by **DQI**.

Parameter	Value
E_T^0	40.80 ± 1.51 kcal mol^{-1}
a	5.18 ± 1.8
b	4.98 ± 2.11
c	–[a]
d	–[a]
r^2	0.751

[a] Discarded parameter during the regression for not contributing to the negative emissive response of **DQI.**

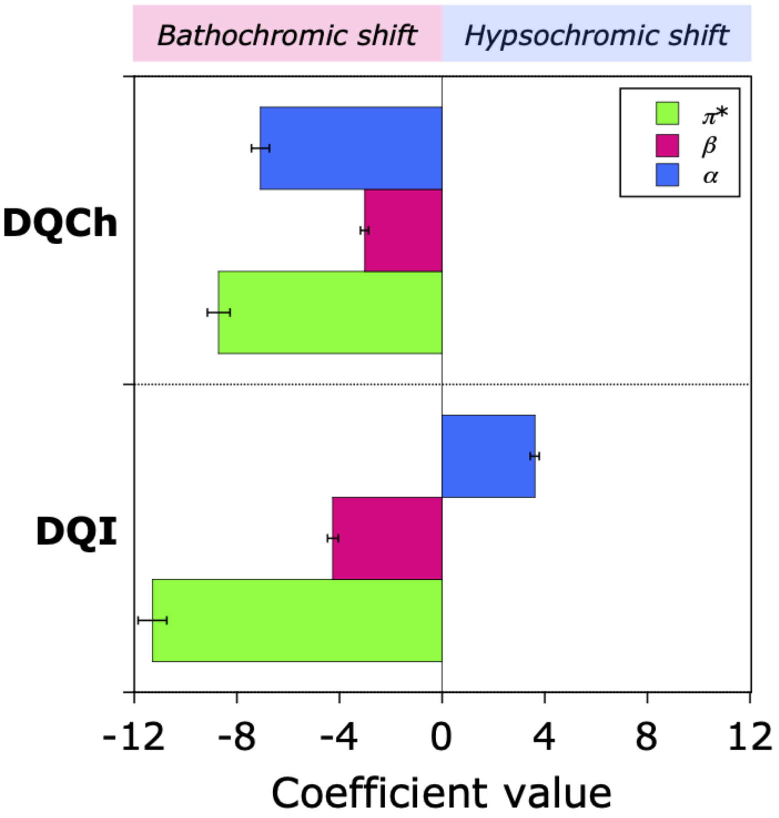

Figure S14. Contribution of KAT parameters dipolarity/polarizability (π^*), hydrogen bond acidity (α), and hydrogen bond basicity (β), to the experimental emissive solvatochromism of DQCh and DQI.

6. Photoisomerization studies of DQI.

When exposed to UV irradiation, substituted alkenes, including **DQCh** and **DQI**, are capable of undergoing *E*/*Z*-isomerization. Therefore, to rule out the possibility of *E*/*Z*-isomerization being involved in the inversion of the emissive solvatochromism demonstrated by **DQI**, its photostability was examined using ^{1}H NMR in CD_3OD, DMSO-d_6, and $CDCl_3$. A LED source was used to irradiate the **DQI** solution in these three deuterated solvents at an intensity of 6440 W/m^2 and a wavelength of λ = 365 nm for periods ranging from 10 s to 24 min. All these results are shown in Figures S15–S17.

As shown in Figure S2, there is no evidence of photodegradation or photoisomerization of **DQI** in CD_3OD. **DQI** is present only in its isomeric *E* form, as evidenced by the signal close to 8.0 ppm that shows a coupling constant of J = 16.3 Hz, and absence of any doublet with J = 12 Hz.

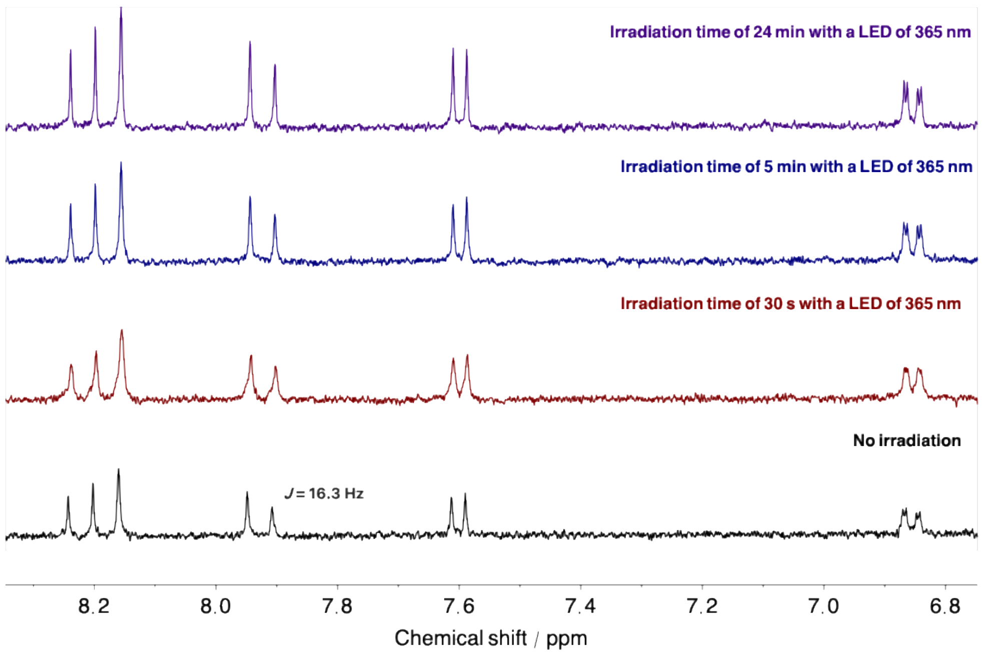

Figure S15. Variation of the ^{1}H NMR signals of **DQI** in a CD_3OD solution at various irradiation times with a LED of λ = 365 nm with an intensity of 6440 W/m^2. The molar concentration of **DQI** was 1.44 mM.

Same situation occurs in DMSO-d_6. As shown in Figure S3, no photodegradation or photoisomerization of **DQI** take place. **DQI** is present only in its isomeric *E* form, as evidenced by the signal close to 7.8 ppm that shows a coupling constant of *J* = 16.1 Hz, and absence of any doublet with *J* = 12 Hz.

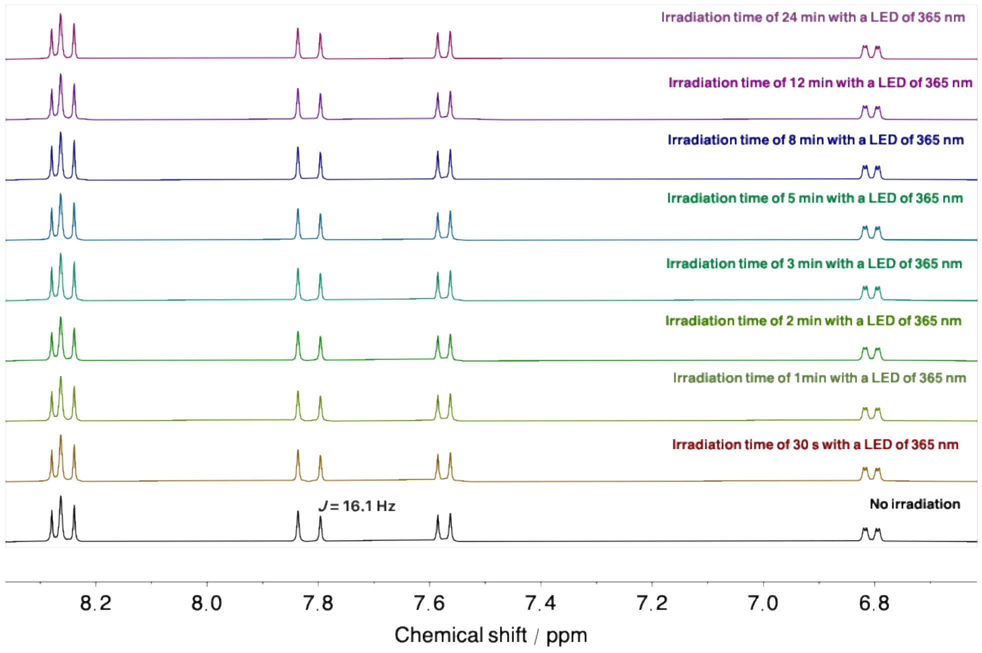

Figure S16. Variation of the ^{1}H NMR signals of **DQI** in a DMSO-d_6 solution at various irradiation times with a LED of λ = 365 nm with an intensity of 6440 W/m^2. The molar concentration of **DQI** was 1.44 mM.

A different situation take place in $CDCl_3$ solution, where **DQI** displays a partial isomerization from the *E*-isomer to the *Z*-isomer. This is evident in the doublet at 8.2 ppm with a coupling constant of *J* = 16.3 Hz, which is present in solution at all irradiation times (5 s to 24 min) and with the doublet at 7.4 ppm with a coupling constant of *J* = 12.8 Hz. This is evidence that in chloroform solution, **DQI** rapidly isomerizes to a maximum *E*/*Z* ratio of 3:1.

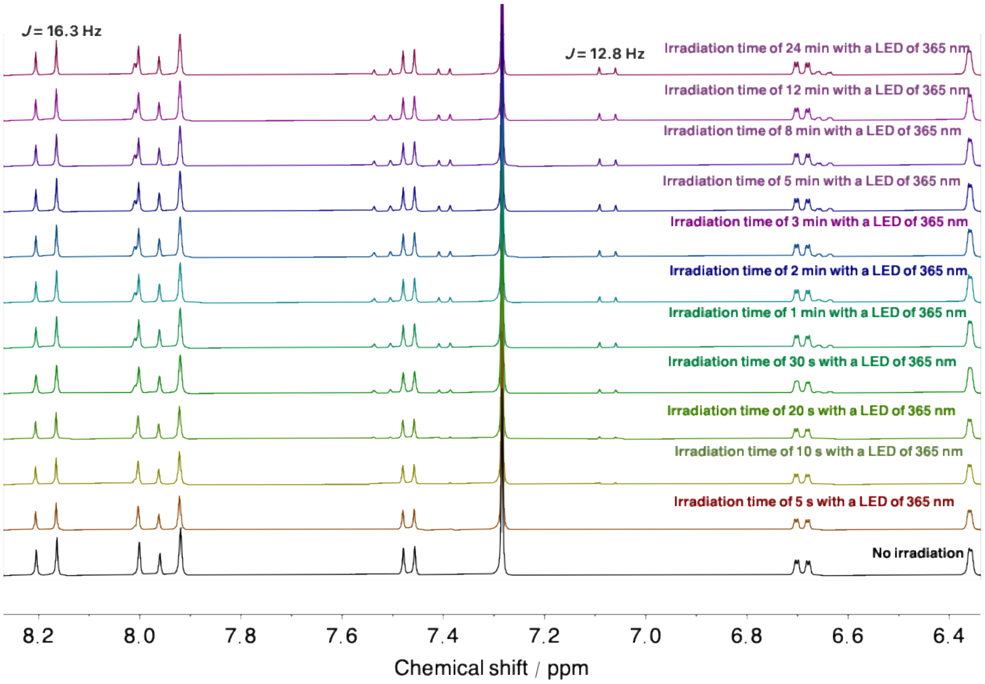

Figure S17. Variation of the ^{1}H NMR signals of **DQI** in a $CDCl_3$ solution at various irradiation times with a LED of λ = 365 nm with an intensity of 6440 W/m^2. The molar concentration of **DQI** was 1.44 mM.

Nevertheless, as shown in Figure S5 the apparition of the *Z*-isomer does not cause any alteration over the emission band of the *E*-isomer located at 621 nm. Therefore, there is no uncertainty about the emission energy of the *E*-isomer reported in the present work.

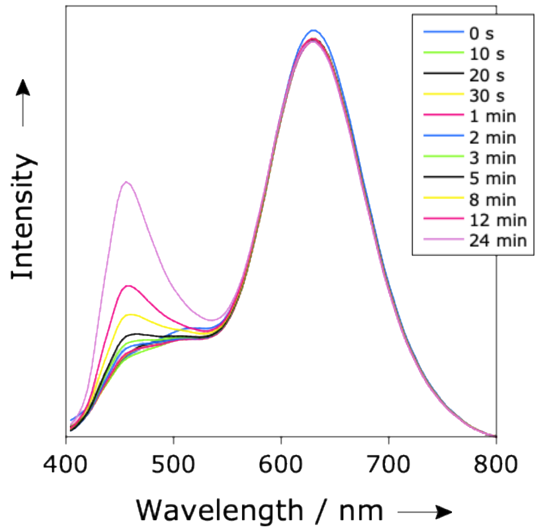

Figure S18. Fluorescence spectra of **DQI** in chloroform solution at room temperature at various irradiation times with a LED of λ = 365 nm with an intensity of 6440 W/m^2.

7. XYZ-coordinates for the optimized ground-state structures of DQI and DQCh, and the optimized excited state structure of DQI.

XYZ-coordinates for the optimized ground-state structure of ***DQCh***

51
Coordinates from ORCA-job opt

C	2.53976282192339	-0.26956869067636	-0.05538254478072
C	3.86204125603004	-0.70892380160280	-0.09883573235503
C	4.93778560729687	0.19532484471427	0.03407343218672
N	6.23049905062239	-0.23872165468053	-0.01615022544704
C	6.56843424003270	-1.62954802320151	-0.31623864123660
C	6.57890154458947	-2.52227103659396	0.92448242636302
C	7.36297846078916	0.65313492409418	0.23907018368102
C	7.85075607333786	1.36988117308894	-1.01889481360057
C	4.63791749345925	1.57806865186593	0.21796573260689
C	3.33656871753776	2.00154082903686	0.26333857645736
C	2.25099993110440	1.11027728867459	0.13108340280355
C	0.91413037344660	1.52783235913184	0.17622184316862
C	-0.14679437042554	0.65365878086431	0.05074332717195
C	-1.48315660444637	1.17861339313976	0.10902657315173
C	-2.67147660274006	0.53613496499731	0.00325476533565
C	-3.92011626788449	1.30157231932293	0.08014820482940
C	-5.21224489688887	0.54724653739135	0.03754255580065
C	-5.30437393166456	-0.82804810997184	0.28802655255351
C	-6.53386569258518	-1.47643504718179	0.26388243589480
C	-7.66365270841513	-0.72983643322742	-0.02542603752016
F	-8.86263447401272	-1.35585401990191	-0.06087474257429
C	-7.61819805110327	0.63318626361523	-0.27913067244825
C	-6.38449873888378	1.26459946648625	-0.23756720681722
O	-3.94108902805851	2.53389819149817	0.16024677316683
C	0.12145762759694	-0.77291529439849	-0.13324168834120
O	-0.75810873004177	-1.62883526231913	-0.24419979890828
N	1.48078976030770	-1.15296852729411	-0.17954448757155
C	1.76733761124293	-2.57547030740722	-0.37112221975561
H	4.06529073333185	-1.76099319870264	-0.21310770845270
H	5.87869959783108	-2.01898088300672	-1.07276825135049
H	7.56044861339963	-1.62968945753446	-0.78089571452936
H	6.85022564788286	-3.54646510091229	0.65038864548293

H	5.59736569295992	-2.54165748233764	1.40641376081616
H	7.30870668168120	-2.15873586224488	1.65437961254811
H	8.17011304617963	0.03994753604866	0.65482636111821
H	7.09623926390817	1.37158307248256	1.02021302642750
H	8.15615827991769	0.64650434894361	-1.78112849890911
H	7.06321266252645	1.99900594622286	-1.44335523983338
H	8.71153043195523	2.00355796091241	-0.78368653081682
H	5.43025768469010	2.30905304025783	0.30261913019184
H	3.11965887370002	3.05750928658961	0.39691516894846
H	0.70736540413914	2.58674822425510	0.31656127014171
H	-1.53670570703295	2.25690661389871	0.26127452764633
H	-2.69946042687778	-0.53028964280183	-0.15928791882848
H	-4.41968895041076	-1.40682892265000	0.52658270315934
H	-6.62198369951102	-2.53806101867994	0.46583294403411
H	-8.53154105639788	1.17372756119783	-0.50180735950024
H	-6.30763147464915	2.33041164914344	-0.42190352192909
H	0.81883674770986	-3.09995809141099	-0.46166330976124
H	2.32247204496238	-2.97089974727565	0.48608691122852
H	2.35657407453707	-2.72306536866045	-1.28211264864752

XYZ-coordinates for optimized ground-state structure of ***DQI***

50

C	-3.65409800165257	-1.82710104546940	-0.12983225318529
C	-4.45826583486501	-0.65073797828256	-0.04218968948645
N	-5.81760313596128	-0.73531943756097	-0.04043760625770
C	-6.52283812597606	-1.99973079235783	-0.26168656583908
C	-6.76731151994608	-2.77744517807682	1.03023335620560
C	-6.66742408651414	0.43659471992466	0.17439215810812
C	-6.97971479939081	1.19118913393580	-1.11735276567751
C	-3.80788097796881	0.60032700513523	0.04668615440904
C	-2.41890506589494	0.69175625165896	0.05287536059722
N	-1.77777373050197	1.91613451648906	0.13297405865412
C	-2.58277608589884	3.13546271048696	0.22113565393736
C	-0.37805823415801	2.06967686210106	0.13427498245712
O	0.12535248728008	3.19099773036277	0.20379608798286
C	0.41433251401345	0.83803605268677	0.05072729726587
C	1.83777794390583	0.99769655023281	0.05872231277295
C	2.75691710496127	-0.00652266742611	-0.00632603796887
C	4.15414385282330	0.23225890841091	0.00742017422144
C	5.27045038546112	-0.60193649668590	-0.04311651838038
N	5.27615781278651	-2.01084442567063	-0.12545723490041
O	6.37285276763167	-2.58192040224415	-0.15695295865649
O	4.19109993870887	-2.60576821525566	-0.16178255116084
C	6.41159160346445	0.25339743114997	0.00923883461913
N	6.03436476203018	1.50372766733979	0.08479291158651
O	4.61233851111438	1.49622840512742	0.08396637521640
C	-0.22925506661955	-0.38025360739543	-0.03100028640057
C	-1.62361607841997	-0.48745646966785	-0.03576530528130
C	-2.28991590216315	-1.72998147865421	-0.12554803469780
H	-4.11126261596598	-2.80580769323508	-0.17696970262478
H	-7.47968914877418	-1.75550884761701	-0.73611082986562
H	-5.97146903111636	-2.60638419339959	-0.98617942092569
H	-7.31849195267021	-3.69834092434204	0.81650709993336
H	-5.82364902446647	-3.04326942827374	1.51498922634884
H	-7.35609725777201	-2.18243996142374	1.73503473477110
H	-7.59777333009822	0.08209597094939	0.63122320716910
H	-6.19844345108968	1.09700349592845	0.91099086293808
H	-7.49350259310485	0.53957492524430	-1.83063062781583

H	-7.62939871425030	2.04597839597786	-0.90588954589175
H	-6.06607893066507	1.56032259096678	-1.59174113625307
H	-4.39664619660374	1.50211058975559	0.08586429032283
H	-1.90221836507104	3.98129565239127	0.28909544361384
H	-3.21005790939932	3.24268513968273	-0.67012930954026
H	-3.22000229549074	3.10542505180815	1.11105897787331
H	2.18205554870257	2.02557171053086	0.12405016662544
H	2.47639182530818	-1.04930885970387	-0.07099890083487
C	7.85999970434865	-0.07906205456673	-0.01032516456505
H	0.34920924702162	-1.29798673608079	-0.09648657282931
H	-1.68629062161894	-2.63114173479843	-0.18382820847656
H	8.44382438440191	0.84122774396302	0.04871437255775
H	8.11117757683255	-0.73151840119922	0.82951511275975
H	8.11311915189174	-0.61973360895284	-0.92571049623164

*XYZ-coordinates for optimized excited-state structure of **DQCh***

51
Coordinates from ORCA-job tpssh E -1250.642427154744

C	2.54640607119230	-0.24703670734110	-0.09867081120787
C	3.86397841665892	-0.70090762794874	-0.09780982824615
C	4.93680539161459	0.20130632607141	0.02362770689729
N	6.22835288444888	-0.24751971402907	0.02589263142110
C	6.55543808305157	-1.65316588430531	-0.19036077557173
C	6.52065844793237	-2.46769906412327	1.10520356898886
C	7.36387879460970	0.64287782857873	0.25069504724050
C	7.88485596737308	1.26413691208037	-1.04664598548036
C	4.65484706228608	1.59251964288764	0.14082881587363
C	3.35333097638457	2.03040817150253	0.13890321746230
C	2.27045408034955	1.13988365330296	0.02321366434160
C	0.91609100861565	1.57882333388811	0.03725327728694
C	-0.13948150271003	0.71239799260272	-0.05868992855326
C	-1.49701278055392	1.23727080722836	-0.00344851149584
C	-2.67185877906813	0.57277959787309	-0.10279599729513
C	-3.95271709039657	1.30012590154307	0.01742271133470
C	-5.19652546224657	0.54096485624137	0.02186783123015
C	-5.27688803063063	-0.86307420312996	-0.14242823086166
C	-6.49376279828374	-1.52379443110057	-0.13274308825342
C	-7.65651120640636	-0.78401638539569	0.04967842747528
F	-8.85444747129552	-1.43431396099048	0.06340727988042
C	-7.63409160538495	0.59435729853121	0.21736561134784
C	-6.41362951593231	1.24619368991272	0.20028370673237
O	-3.94961029560049	2.55365587684140	0.13840335037920
C	0.13949953932300	-0.72862991656317	-0.19490398904564
O	-0.73789624029183	-1.58178120933823	-0.29568128973910
N	1.48726727891629	-1.12263392604562	-0.20708087934417
C	1.76302373405332	-2.55230026064280	-0.33576366318526
H	4.05895087873842	-1.75693134310745	-0.16921526070095
H	5.87374022921808	-2.06874467633079	-0.93280126388991
H	7.55709921511838	-1.68035269975781	-0.62140592539341
H	6.78918044939978	-3.50400071093543	0.89288367738210
H	5.52590631350807	-2.44887711569864	1.55179458005143
H	7.23190026431625	-2.06752250932097	1.82950190229966
H	8.14518708454166	0.04093912530369	0.71744757969755
H	7.08134298759319	1.41247377886233	0.96788875269896

H	8.19100495201971	0.48813413716420	-1.75015736668902
H	7.11590324857848	1.87606055471122	-1.51990714596438
H	8.74940735860397	1.89387952607636	-0.82946537699218
H	5.45556550282232	2.31211673828079	0.21611567096209
H	3.14275399501443	3.08937526798041	0.22451938462505
H	0.72135307139225	2.64073217325101	0.13712417983003
H	-1.55908092497516	2.31192393953344	0.14300421918725
H	-2.66974373261186	-0.49454691712008	-0.25360176599960
H	-4.38068842978780	-1.45132326426608	-0.28536184572897
H	-6.55146824473290	-2.59708482814441	-0.26199686950740
H	-8.56173550591342	1.13561655809989	0.35656090122539
H	-6.36232918254336	2.31955998586284	0.32528615435469
H	0.80981462950228	-3.06121570698587	-0.42047925286423
H	2.29761820920664	-2.91011961426850	0.54569741062710
H	2.36415731158164	-2.73601675412186	-1.22758087582388

8. Solvent effect on the spectral shifts of DQCh and DQI

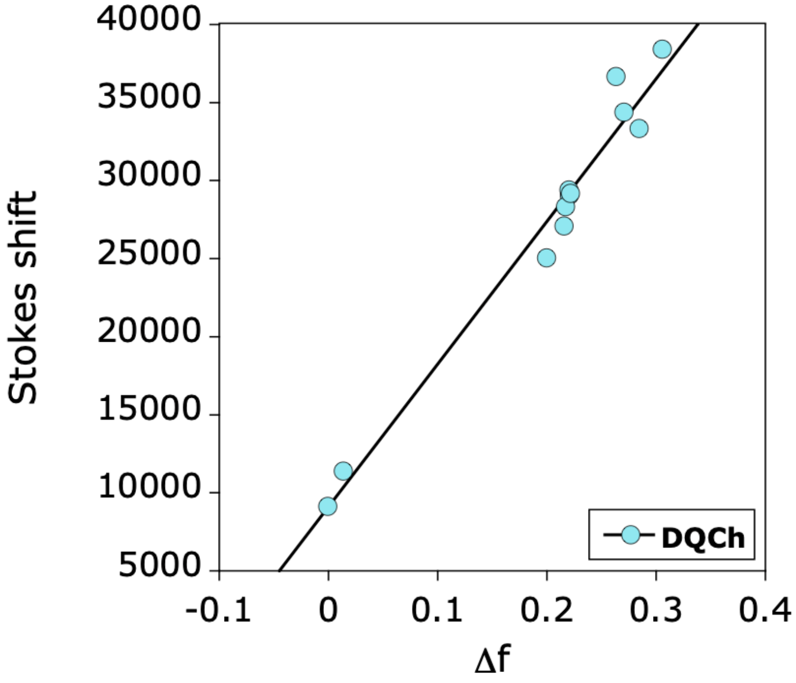

Figure S19. Lippert-Mataga plot of **DQCh** dye.

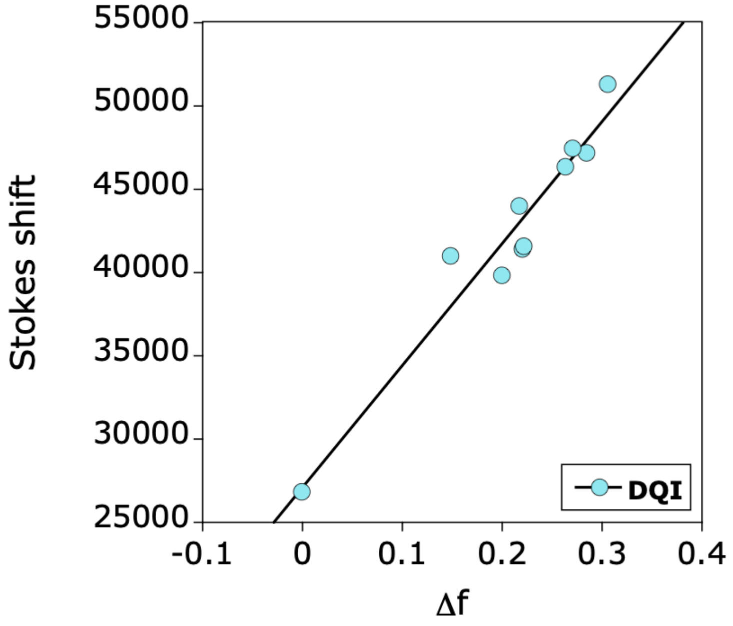

Figure S20. Lippert-Mataga plot of **DQI** dye.
